# Screening for patients at risk for cardiac amyloidosis via electronic health records: A multicenter machine learning development and validation study

**DOI:** 10.1371/journal.pdig.0001637

**Published:** 2026-10-08

**Authors:** Clemens P. Spielvogel, David Kersting, David Haberl, Maximilian Autherith, Laurenz Hauptmann, Josef Yu, Juliane Hennenberg, Kilian Kluge, Kim Moon, Stephan Settelmeier, Jing Ning, Katarina Kumpf, Markus Köfler, Felix Hofer, Katharina Mascherbauer, Julia Mascherbauer, Andreas Kammerlander, Tatjana Traub-Weidinger, Gregor Kasprian, Tienush Rassaf, Jens Kleesiek, Ken Herrmann, Philipp Bartko, Christian Hengstenberg, Marcus Hacker, Raffaella Calabretta, Christian Nitsche

**Affiliations:** 1 Division of Nuclear Medicine, Department of Biomedical Imaging and Image-guided Therapy, Medical University of Vienna, Vienna, Austria; 2 Comprehensive Center for Artificial Intelligence in Medicine, Medical University of Vienna, Vienna, Austria; 3 Department of Nuclear Medicine, University Hospital Essen, University of Duisburg-Essen, Essen, Germany; 4 Christian Doppler Laboratory for Applied Metabolomics, Medical University of Vienna, Vienna, Austria; 5 Division of Cardiology, Department of Internal Medicine II, Medical University of Vienna, Vienna, Austria; 6 Division of Neuroradiology and Musculoskeletal Radiology, Department of Biomedical Imaging and Image-guided Therapy, Medical University of Vienna, Vienna, Austria; 7 Institute for Artificial Intelligence in Medicine, University Hospital Essen, Essen, Germany; 8 Department of Cardiology and Vascular Medicine, West German Heart and Vascular Center, University Hospital Essen, Essen, Germany; 9 IT4Science, IT Services & Strategic Information Management, Medical University of Vienna, Vienna, Austria; 10 Department of Internal Medicine 3, University Hospital St. Pölten, St. Pölten, Karl Landsteiner University of Health Sciences, Krems an der Donau, Austria; 11 Division of Diagnostic and Therapeutic Nuclear Medicine, Donau Hospital Vienna, Vienna, Austria; 12 West German Amyloidosis Center, University Hospital Essen, Essen, Germany; University of Cambridge, UNITED KINGDOM OF GREAT BRITAIN AND NORTHERN IRELAND

## Abstract

Timely detection is crucial to improve outcomes in patients with cardiac amyloidosis (CA) by initiation of guideline-directed disease-modifying treatments. Although confirmatory bone scintigraphy is highly accurate for CA detection, identifying at-risk patients for referral remains challenging. This study aimed to develop and validate a machine learning model, *Amylo-Detect*, using structured multimodal electronic health record (EHR) data to guide referrals for confirmatory scintigraphy and monoclonal protein testing. Consecutive all-comer patients (n = 11,616) referred for bone scintigraphy at the Vienna General Hospital (2010–2023) were retrospectively included. Patients referred before August 2020 formed the development cohort. The remaining patients comprised the internal validation cohort. External validation was performed at the University Hospital Essen (n = 1,521). *Amylo-Detect* was trained using 50 routinely available parameters to predict CA-suggestive uptake (Perugini grade ≥2) and compared with an existing score and clinical routine. High-grade uptake was present in 388 patients (3.0%). *Amylo-Detect* demonstrated excellent performance in development (AUC 0.93), independent internal validation (AUC 0.91), and external validation cohort (AUC 0.91), outperforming existing scoring systems and clinical routine. Results were consistent across subgroups, even when crucial predictors were missing. Of the 42/388 (10.8%) patients missed in clinical routine, 12/42 (29%) were additionally detected by *Amylo-Detect*. The model further conveyed significant prognostic value for mortality and heart failure hospitalization. We present *Amylo-Detect*, a validated EHR-based tool for CA risk prediction, available as a web app, allowing application and further evaluation. By improving timely detection and referral, *Amylo-Detect* may help address diagnostic delays, pending prospective evaluation.

## Author summary

Cardiac amyloidosis is a progressive and often fatal heart disease that is frequently diagnosed too late, even though effective treatments are now available. A major challenge is recognizing which patients should be referred for specialized testing, because early symptoms are diverse and often non-specific. In this study, we developed and validated a machine learning prediction system, called *Amylo-Detect*, that uses routinely collected information from electronic health records to identify patients who may be at risk for cardiac amyloidosis. We trained and externally validated the tool using data from 13,137 patients across two hospitals. We found that *Amylo-Detect* was highly accurate in identifying patients with disease-indicative findings on confirmatory scans and identified a substantial number of patients who were not initially suspected of having cardiac amyloidosis. *Amylo-Detect*, which we make publicly available via a web app, consistently outperformed existing risk scores and routine clinical decision-making. Our findings suggest that automated analysis of electronic health records can support clinicians in recognizing cardiac amyloidosis earlier, reduce diagnostic delays, and potentially improve patient outcomes by enabling timely treatment.

## Introduction

Cardiac amyloidosis (CA) is a progressive and fatal disease characterized by misfolded proteins aggregating to amyloid fibrils with subsequent deposition in the myocardium, leading to heart failure and death. The two major types of CA include transthyretin (ATTR) and light chain (AL) amyloidosis, making up for more than 98% of currently diagnosed CA cases [1]. Over the last decade ATTR-CA in particular has emerged as substantially more common than previously appreciated, accounting for approximately 6–13% of patients with heart failure with preserved ejection fraction [2,3], 13% of elderly patients with severe aortic stenosis [4], and 1.5% (Perugini grade≥2) to 3.3% (Perugini grade≥1) among all-comer patients undergoing bone scintigraphy [5]. However, despite considerable advances regarding disease awareness, CA is still considered underdiagnosed and the early and accurate identification of patients remains a major challenge, particularly as disease-modifying treatments are believed to be most effective in early disease stages [6–8]. A critical bottleneck is raising the initial suspicion of CA early and accurately to refer patients to confirmatory testing by scintigraphy and monoclonal protein testing.

Amyloidosis is a systemic disease and can manifest through a diverse array of symptoms such as neuropathy, renal dysfunction, and gastrointestinal issues. These indicators are non-specific for CA when observed in isolation. However, the presence of multiple indicators can help to raise an initial suspicion. This multi-symptomatic presentation makes CA an ideal target for data-driven, multimodal approaches that can integrate routinely collected parameters and uncover complex, non-linear patterns that predict CA.

Prior artificial intelligence (AI) efforts in amyloidosis have shown promise when applied to cohorts with a high pretest probability [9,10]. However, such methods typically do not generalize when deployed across all-comer patients where the prevalence is low and the clinical presentation heterogeneous, limiting their applicability in clinical practice.

In this study, we present *Amylo-Detect*, a fully automated machine learning tool developed and validated using routinely available pre-scintigraphy parameters from electronic health records (EHR) of consecutive, all-comer patients referred for bone scintigraphy and independently validated at two sites. *Amylo-Detect* predicts CA-suggestive tracer uptake (Perugini grade ≥2) to guide referral for confirmatory testing. This approach aims to close diagnostic gaps in CA by accelerating the identification of at-risk patients, enabling timely confirmatory testing and treatment to ultimately improve cardiovascular outcomes.

## Methods

### Study design and participants

This large-scale multicenter cohort study included consecutive all-comer referrals for whole-body ^99m^Tc-3,3-diphosphono-1,2-propanodicarboxylic acid (DPD) scintigraphy at the Vienna General Hospital (Austria) between January 2010 and February 2023. Patients examined before August 2020 were included for development and the remaining patients for independent internal validation (S1 Table and S2 Table). For external validation, all-comer referrals for DPD scintigraphy were collected at the University Hospital Essen (Germany), acquired between August 2018 and April 2025 (S3 Table). Beyond scintigraphy images, collected data included corresponding baseline parameters from EHR, including demographic, blood, echocardiography, and comorbidities. For external validation, the six most important parameters were collected to enable model inference. To reflect a realistic clinical routine scenario, patients with missing values in baseline parameters were retained to ensure clinical applicability. Tricuspid, pulmonary, mitral, and aortic valve parameters included stenosis as well as regurgitation and were dichotomized into moderate or higher and less than moderate. All-cause mortality and heart failure-associated hospitalization (HFH) were assessed in the Vienna cohorts and were 100% and 92% complete, respectively. All-cause mortality was captured via the Austrian Death Registry. HFH was determined from patient records of the Vienna General Hospital, the Vienna-Health-Association database, and the nationwide EHRs. This study received approval from the Medical University of Vienna’s institutional review board (1557/2020) and was carried out in compliance with the principles of the 1964 Declaration of Helsinki. Owing to the retrospective design of the study, the requirement for informed consent was waived.

### Image acquisition and annotation

Image acquisition was performed using standardized institutional routine protocols for ^99m^Tc-DPD whole-body scintigraphy with post-injection acquisition times of ≥2 hours in all cohorts. Cardiac tracer uptake was annotated according to the Perugini classification [11,12], whereby grade 0 represents no cardiac uptake, grade 1 minor cardiac uptake (heart less than bone) and grade 2 or 3 intense cardiac uptake (heart equal to or greater than bone). Grade 2 or higher uptake in the absence of monoclonal light chains is highly specific and sensitive for cardiac amyloidosis. Grading was therefore dichotomised into high-grade (cardiac-amyloidosis-suggestive uptake: grade 2 or 3) and non-suggestive uptake (grade 0 or 1). For the Vienna cohorts, grading was performed by a centralized core laboratory at the Medical University of Vienna comprising three independent, mutually blinded specialists with at least 5 years of experience (CN, TTW and JN). Discrepancies, which occurred almost exclusively at the grade 1 versus grade 2 border, were resolved by consensus reading by two further specialists with at least 10 years of experience (RC and Alexander R. Haug). At the external site, Perugini grades were annotated based on the clinical routine imaging reports and stratified into high-grade (Perugini grade ≥2) or non-suggestive uptake (Perugini grade ≤1).

### Diagnostic model for prediction of CA-suggestive uptake

Machine learning was performed to predict high-grade cardiac uptake on scintigraphy indicative of CA using the 50 baseline parameters. The machine learning process consisted of model development and cross-validation, followed by independent internal validation using data from a different time range and external validation. Stratified 10-fold cross-validation was performed on the development cohort. Subsequently, the *Amylo-Detect* model was built on the entire development dataset and validated on the independent internal and external validation datasets. During cross-validation, each fold’s preprocessing included the standardization of features, imputation using k-nearest neighbors, selection of features through the minimum redundancy maximum relevance (mRMR) algorithm [13], addressing imbalances with the synthetic minority oversampling technique (SMOTE) [14], and automated hyperparameter tuning via a random search within a pre-defined hyperparameter grid. Missing values were imputed using k-nearest-neighbors imputation (k = 10, Euclidean distance, distance weighting using inverse distance) fitted exclusively on the training data and then applied unchanged to the validation cohorts. No explicit missingness-indicator variables were added as missingness in routine records may encode local workflow, investigation availability and referral practice rather than disease biology. Predictions in patients with extensive missingness should be interpreted with corresponding caution. For feature selection, a total of log2(n) features were selected, where n represented the number of total patients in the development cohort. Additionally, features were removed if their normalized logistic regression coefficients were below 0.01 to avoid features with minimal effect on the model. Preprocessing algorithms were applied in the aforementioned order. Any kind of fitting procedures during the entire modelling process, including preprocessing steps, were solely performed on the training data to avoid any kind of data leakage. Training was performed using seven machine learning algorithms, including random forest, k-nearest neighbors, extreme gradient boosting, logistic regression, support vector machine, decision tree, and explainable boosting machine. Risk calibration was performed using logistic recalibration, with the recalibration map estimated on the development cohort by maximum likelihood and applied unchanged to both validation cohorts. For binary classification, a cut-off of 0.043 for the continuous probability-like scores was selected in the development data and employed to ensure a reasonable balance between diagnostic metrics. Importantly, by outputting a continuous probability-like score, *Amylo-Detect* allows for the selection of center-specific and clinical context-specific cut-offs. The entire model, including all preprocessing, is publicly available via the free *Amylo-Detect* Web App (https://amylo-detect.streamlit.app/). The web app supports this concept by providing the output as a probability-like score rather than a fixed label. All fitted models, including standardization, imputation, feature selection, hyperparameter tuning, classification modelling, and calibration, were fitted during training and not changed before application to any validation cohorts.

### Comparison with existing risk score and clinical routine

*Amylo-Detect* was evaluated against the Mayo transthyretin amyloid cardiomyopathy (ATTR-CM) score, which has previously demonstrated strong performance in both a pyrophosphate (PYP) scan referral cohort and in patients with heart failure with preserved ejection fraction (HFpEF) [9]. The comparison was conducted in the all-comer cohort and a small subgroup with high pre-test probability, defined as an extracellular volume (ECV) >40% on cardiac magnetic resonance imaging (CMR) [15–17]. To ensure comparability, imputation of missing values required for the calculation of the Mayo ATTR-CM score was performed using the same k-nearest-neighbors imputation procedure used for the machine learning models.

For the comparison of *Amylo-Detect* with clinical routine suspicion, referral indications were collected for all scans. *Amylo-Detect* was compared with the clinical referral indication regarding to the same diagnostic performance metrics based on whether the referred patient was found to have high-grade cardiac uptake. AUC for clinical suspicion was calculated at a single operating point, as the arithmetic mean of sensitivity and specificity. This analysis was performed among patients who were assessed for CA-suggestive uptake via scintigraphy.

### Statistical analysis

Continuous parameters are reported as mean ± standard deviation (SD) or median with interquartile range (IQR). Categorical data are presented as counts and percentages. To compare patient characteristics across groups, numerical variables were analyzed using Mann-Whitney U test or T-test, and categorical variables were evaluated either using Fisher’s exact test or Chi-squared test. The Shapiro-Wilk test was applied to determine normality for selecting the appropriate statistical test. The performance of machine learning models is reported using the area under the receiver operating characteristic curve (AUC), and various other diagnostic metrics with corresponding 95% confidence intervals (CIs). CIs were computed over the ten cross-validation folds in the development cohort and using 1,000 bootstrap samples in the independent internal and external validation cohorts. Model AUCs were compared using the DeLong test. If patients underwent multiple scans, only the first scan was employed to avoid any data leakage or model bias. Kaplan-Meier estimates and Cox proportional hazard models were applied to assess association of model predictions with all-cause mortality and HFH, starting with the day of scintigraphy. Multivariable adjustment was performed for cancer, age, sex, BMI, renal insufficiency, atrial fibrillation, hypertension, diabetes, coronary artery disease, COPD, carpal tunnel syndrome, heart-failure history, and interventricular septum thickness. *P* values less than 0.05 were deemed statistically significant. All developments and analyses were performed using Python 3.9.5. Prior to all analyses, random seeds were set to the default where available, otherwise to zero. This study and the related reporting adhere to the TRUE-AIM guidelines [18].

## Results

### Population characteristics

A graphical overview of the study is shown in Fig 1. The development cohort included 9,170 patients with a median age of 64 (IQR 52–73) years. Patients with high-grade uptake were considerably older than the overall cohort (median age 79 [IQR 71–87] years in development), consistent with predominantly wild-type ATTR-CM in this population. Overall, 5,488/9,170 (60%) patients were female. The separately collected internal validation cohort included 2,446 patients with a median age of 67 (IQR 55–76) years and 1,121/2,446 (46%) females. The external validation cohort comprised 1,521 patients with a median age of 69 (IQR 59–79) years and 882/1,521 (58%) females. High-grade cardiac uptake was present in 142/9,170 (1.5%) of the development cohort, in 101/2,446 (4.1%) of the internal validation and in 145/1,521 (9.5%) patients of the external validation cohort. The number of scans performed with the query of suspected CA was 1,003/9,170 (11%) in the development, 576/2,446 (24%) in the internal validation cohort, and 516/1,521 (33.9%) in the external validation cohort. Detailed referral indications for the Vienna cohorts are shown in S1 Fig. Of 388 patients with high-grade cardiac uptake, 42 (10.8%) were referred for non-cardiac indications. Comprehensive patient characteristics of the development, internal, and external validation cohorts are shown in S1 Table, S2 Table and S3 Table. A study flow diagram is shown in S2 Fig.

**Fig 1:**
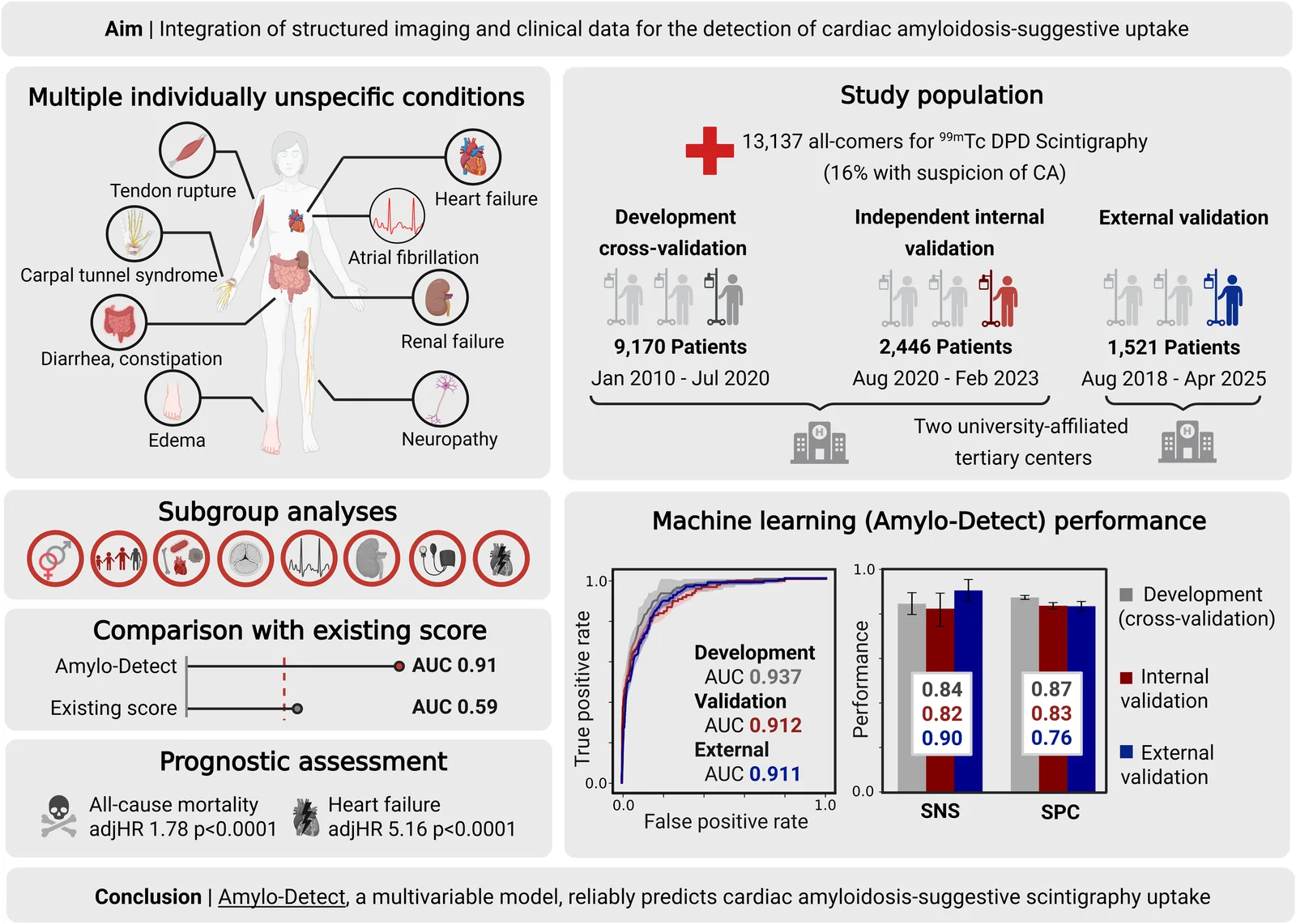
Graphical abstract.

### Diagnostic performance of the machine learning model

A total of 11 features were selected into the final model, including carpal tunnel syndrome, atrial fibrillation, sex, left ventricular diastolic dysfunction, interventricular septum (IVS) thickness and atrial diameter on echocardiography, age, blood urea nitrogen (BUN), history of heart failure, NT-proBNP, and triglycerides (S4 Table). During development, the machine learning-enhanced logistic regression achieved the highest performance with a cross-validated AUC of 0.93 (95% CI 0.91; 0.95) (S3 Fig). Consequently, this model, we named *Amylo-Detect*, was employed for all subsequent analyses. In the independent internal validation, *Amylo-Detect* demonstrated robust performance with an AUC of 0.91 (95% CI 0.88; 0.94). Results remained consistent in external validation with an AUC of 0.91 (95% CI 0.89; 0.93). Comprehensive diagnostic metrics are shown in Table 1. Risk calibration on the development data improved calibration in all three cohorts (S4 Fig). Confusion matrices for all cohorts are shown in S5 Fig. The six parameters with the highest importance for prediction were (uni- or bilateral) carpal tunnel syndrome, followed by atrial fibrillation, sex, left ventricular diastolic dysfunction, interventricular septum thickness on echocardiography as well as patient age.

**Table 1:** Machine learning performance for the prediction of cardiac amyloidosis-indicative uptake on bone scintigraphy.

Approach	Cohort	AUC (95% CI)	ACC (95% CI)	AUPRC (95% CI)	SNS (95% CI)	SPC (95% CI)	PPV (95% CI)	NPV (95% CI)
*Amylo-Detect*	Development (cross-validation)	**0.93** **(0.91; 0.95)**	**0.93 (0.92; 0.93)**	**0.31 (0.24; 0.39)**	0.71 (0.63; 0.79)	**0.93 (0.92; 0.93)**	**0.14 (0.12; 0.15)**	1.00 (0.99; 1.00)
Clinical routine	Development (cross-validation)	0.86 (0.84; 0.88)	0.88 (0.87; 0.88)	0.11 (0.10; 0.12)	**0.84 (0.80; 0.89)**	0.88 (0.87; 0.89)	0.13 (0.12; 0.14)	1.00 (0.99; 1.00)
*Amylo-Detect*	Independent internal validation	**0.91 (0.88; 0.94)**	**0.93 (0.92; 0.94)**	**0.54 (0.45; 0.63)**	0.64 (0.55; 0.74)	**0.94 (0.93; 0.95)**	**0.32 (0.28; 0.37)**	0.98 (0.98; 0.99)
Clinical routine	Independent internal validation	0.84 (0.80; 0.87)	0.79 (0.78; 0.81)	**0.14 (0.12; 0.16)**	**0.88 (0.81; 0.94)**	0.79 (0.77; 0.81)	0.15 (0.14; 0.17)	**0.99 (0.99; 1.00)**
*Amylo-Detect*	External validation	**0.91** **(0.89; 0.93)**	**0.87 (0.85; 0.89)**	**0.56 (0.48; 0.63)**	0.73 (0.66; 0.80)	**0.88 (0.87; 0.90)**	**0.39 (0.35; 0.44)**	0.97 (0.96; 0.98)
Clinical routine	External validation	0.85 (0.83; 0.87)	0.75 (0.73; 0.77)	**0.27 (0.25; 0.29)**	**0.97 (0.94; 0.99)**	0.73 (0.70; 0.75)	0.27 (0.26; 0.29)	**1.00 (0.99; 1.00)**

AUC, area under the receiver operating characteristic curve; SNS, sensitivity; SPC, specificity; PPV, positive predictive value; NPV, negative predictive value; Bold values indicate higher performance for each comparison.

### Comparison with existing risk score

The *Amylo-Detect* model demonstrated superior performance in the identification of at-risk patients for CA compared to the previously published Mayo ATTR-CM score in the all-comer cohort for all diagnostic metrics, including AUC (all *p* < 0.01). In the high pre-test probability cohort with a CMR-ECV > 40%, the Mayo ATTR-CM score demonstrated substantially improved performance compared to the all-comer cohort. Nevertheless, the *Amylo-Detect* model continued to outperform the Mayo ATTR-CM score (S5 Table).

### Comparison with routine clinical suspicion among patients who underwent scintigraphy

The routine clinical suspicion for the referral of patients for scintigraphy due to the suspicion of CA yielded an AUC of 0.86 (95% CI 0.84; 0.88), 0.84 (95% CI 0.80; 0.87), and 0.85 (95% CI 0.83; 0.87) for cross-validation, internal validation and external validation cohorts, respectively. *Amylo-Detect* outperformed the clinical standard in all but 1 or 2 metrics, depending on the cohort (Table 1). At an operating point of 0.043, sensitivity is lower than clinical routine (0.71 vs 0.84 development, 0.64 vs 0.88 internal validation, 0.73 vs 0.97 external validation), while specificity and PPV are consistently higher (specificity 0.93 vs 0.88, 0.94 vs 0.79, 0.88 vs 0.73; PPV 0.14 vs 0.13, 0.32 vs 0.15, 0.39 vs 0.27). The threshold is set to favor rule-in efficiency. When applied in a clinical routine scenario, as the model supplements rather than replaces clinical suspicion, the operationally relevant sensitivity is that of model plus routine combined, which exceeds either alone. A direct comparison for AUC cannot be made since clinical suspicion only allows measurement at a single operating point. 12/42 (29%) of the patients missed in clinical routine were identified by *Amylo-Detect*. Incidental detection of CA-suggestive uptake was significantly associated with worse outcome, highlighting the clinical importance of such findings. Among patients with CA-suggestive uptake missed in clinical routine referral, patients had markedly lower bilirubin values (p = 0.046), were less commonly affected by carpal tunnel syndrome (p = 0.03) and had significantly lower hemoglobin (p = 0.02) and hematocrit (p = 0.049) values in comparison with patients correctly identified by routine clinical suspicion. Other clinical characteristics were comparable between groups.

### Subgroup and robustness analyses

Subgroup analyses were performed for the comprehensively annotated Vienna cohorts across prespecified subgroups and demonstrated robust performance (Fig 2). This included subgroups where underlying or concomitant CA may be masked by conditions commonly associated with left ventricular thickening. We further performed subgroup analyses for patients with/without carpal tunnel syndrome and available echocardiography data which may sometimes be available after an initial suspicion was raised clinically. In both subgroups, *Amylo-Detect* demonstrated robust performance (AUC 0.88-0.96).

**Fig 2:**
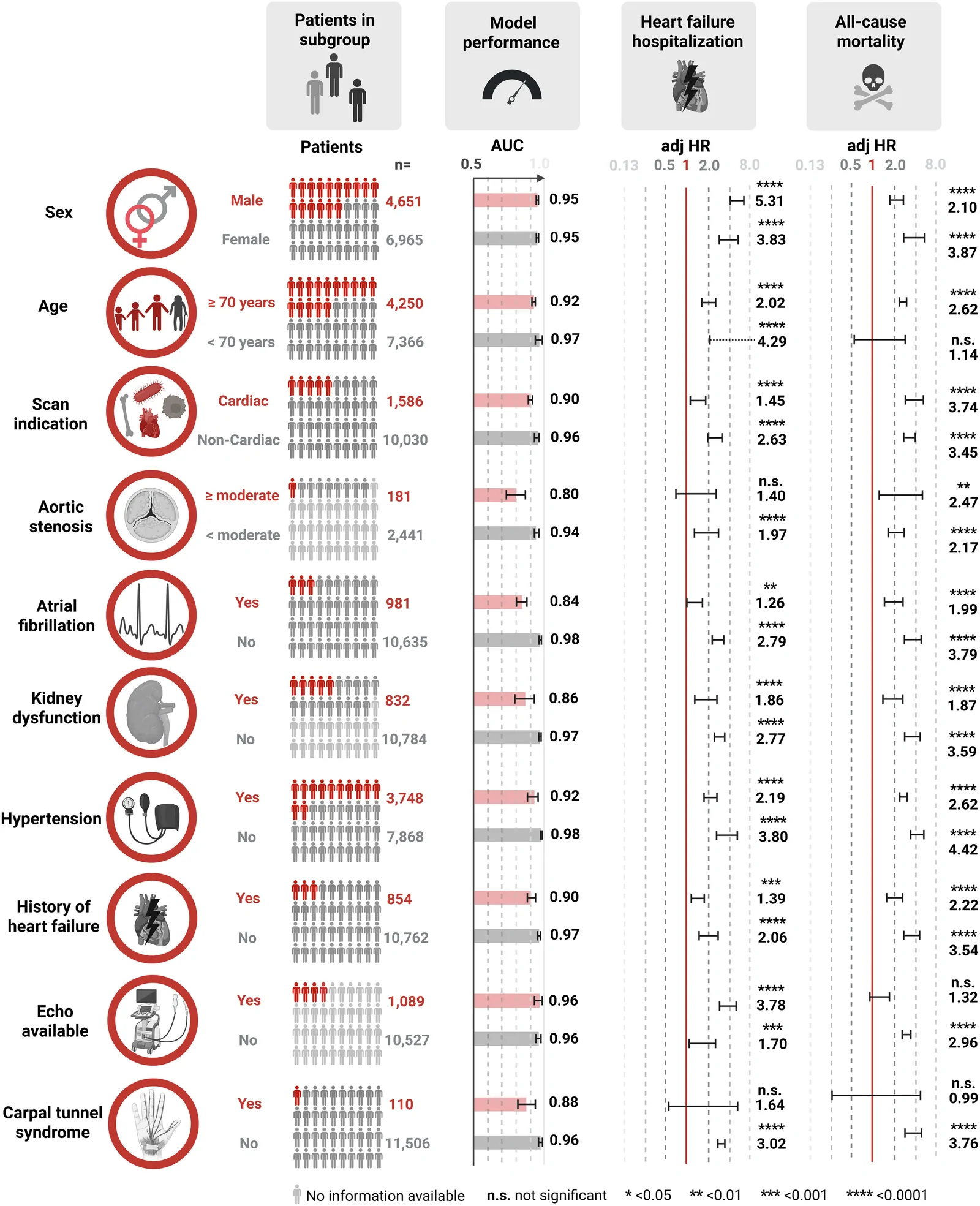
Subgroup analysis results. Cox proportional hazard models for heart failure-associated hospitalization and all-cause mortality were performed based on the prediction by *Amylo-Detect* within the respective subgroups.

On average, patients had 38 missing parameters (IQR 33–46), 6 (IQR 5–7) of which were among the 11 selected parameters in the model. The model demonstrated robustness for missing values and yielded even slightly higher performance for patients with more than 6 missing values, among the selected parameters, compared to patients with fewer missing values (AUC 0.94 [95% CI 0.92; 0.96] vs. AUC 0.91 [95% CI 0.89; 0.93], p < 0.05).

We further performed an analysis of patients with high-grade cardiac uptake and confirmed CA subtype - either based on biopsy with mass spectrometry or based on single-photon emission computed tomography/computed tomography (SPECT/CT) and light chain assessment. Among the 62 patients included, there were 18 patients with biopsy-confirmed ATTR-CA, 17 (94%) of which were identified as at-risk by *Amylo-Detect*. Of the 3 patients with biopsy-confirmed AL-CA, 2 (67%) were predicted correctly. There were 2 patients with combined ATTR-CA and AL-CA, all (100%) of whom were correctly predicted by *Amylo-Detect*. Among the remaining 39 SPECT-confirmed patients, 31 (79%) were correctly identified.

At a sensitivity-prioritized threshold (0.014), defined by lowering the cut-off until sensitivity reached 0.90 in the development cohort, Amylo-Detect maintained high sensitivity across all cohorts (development 0.90, 95% CI 0.85 to 0.94; internal validation 0.86, 95% CI 0.79 to 0.93; external validation 0.94, 95% CI 0.90 to 0.97). Negative predictive value remained close to optimal (development 1.00, 95% CI 1.00 to 1.00; internal validation 0.99, 95% CI 0.99 to 1.00; external validation 0.99, 95% CI 0.99 to 1.00). As expected, given the low prevalence of high-grade uptake (3.0%), positive predictive value at a screening threshold was modest (development 0.08, 95% CI 0.07 to 0.08; internal validation 0.14, 95% CI 0.13 to 0.16; external validation 0.25, 95% CI 0.24 to 0.27), corresponding to approximately 4–13 confirmatory scans per additional case identified. Specificity at this operating point was 0.83 (95% CI 0.82 to 0.83), 0.78 (95% CI 0.76 to 0.79) and 0.71 (95% CI 0.68 to 0.73), respectively.

Lastly, a sensitivity analysis was performed to evaluate the detection of patients with any cardiac uptake (Perugini grade ≥1), using the same model trained to detect Perugini grade *≥2* cardiac uptake. The model achieved an AUC of 0.86 (95% CI 0.85; 0.88) in the development cohort, AUC 0.90 (95% CI 0.87; 0.92) in the independent internal validation cohort and AUC 0.94 (95% CI 0.91; 0.96) in the external validation cohort (S6 Fig). We further show performance metrics over various thresholds in S7 Fig.

### Clinical utility

To assess clinical usefulness beyond discrimination, we performed decision-curve analysis and quantified the downstream testing burden at the operating point (threshold 0.043).

Decision-curve analysis (S8 Fig) showed that the net benefit of *Amylo-Detect* at the operating point exceeded that of clinical routine from a threshold probability of approximately 0.06 in development and 0.07 in internal validation. In external validation, where routine suspicion was highly sensitive (0.97) but poorly specific (0.73), net benefit of *Amylo-Detect* exceeded that of clinical routine from a threshold probability of approximately 0.14 upward.

At the operating point, the number needed to scan to identify one patient with high-grade uptake was 7.1, 3.1, and 2.6 in development, internal validation, and external validation, compared with 7.7, 6.7, and 3.7 for clinical routine. False positives per 1000 patients screened were 69, 58, and 109 for *Amylo-Detect* versus 118, 201, and 244 for clinical routine. The number needed to screen to identify one case was 88, 38, and 14 across the three cohorts. In external validation, application of *Amylo-Detect* corresponded to approximately 178 patients per 1000 referred for confirmatory scintigraphy and monoclonal protein testing, compared with 336 per 1000 under clinical routine, at a sensitivity of 0.73 versus 0.97.

### Prognostic value

To assess the prognostic value of the model’s predictions, their association with clinical outcomes was evaluated in both the cross-validation and internal validation cohorts (Fig 3). After a median follow-up of 66 months (IQR 35–103) after scintigraphy, 590/11,342 (5.2%) patients experienced HFH and 2,877/11,616 (24.8%) patients died. In the cross-validation cohort, predictions were strongly associated with all-cause mortality (adjHR 3.49 [95% CI 3.08; 3.95], p < 0.0001) and HFH (adjHR 5.73 [95% CI 4.67; 7.04], p < 0.0001). These associations remained significant in the internal validation cohort, both for all-cause mortality (adjHR 2.00 [95% CI 1.42; 2.81], p = 0.0007) and HFH (adjHR 4.98 [95% CI 3.18; 7.79], p < 0.0001). Among patients with referral indications other than CA, incidental high-grade cardiac uptake was significantly associated with all-cause mortality (adjHR 3.47 [95% CI 1.52; 7.92], p < 0.0001) and HFH (adjHR 1.70 [95% CI 1.09; 2.66], p = 0.02) (S9 Fig).

**Fig 3:**
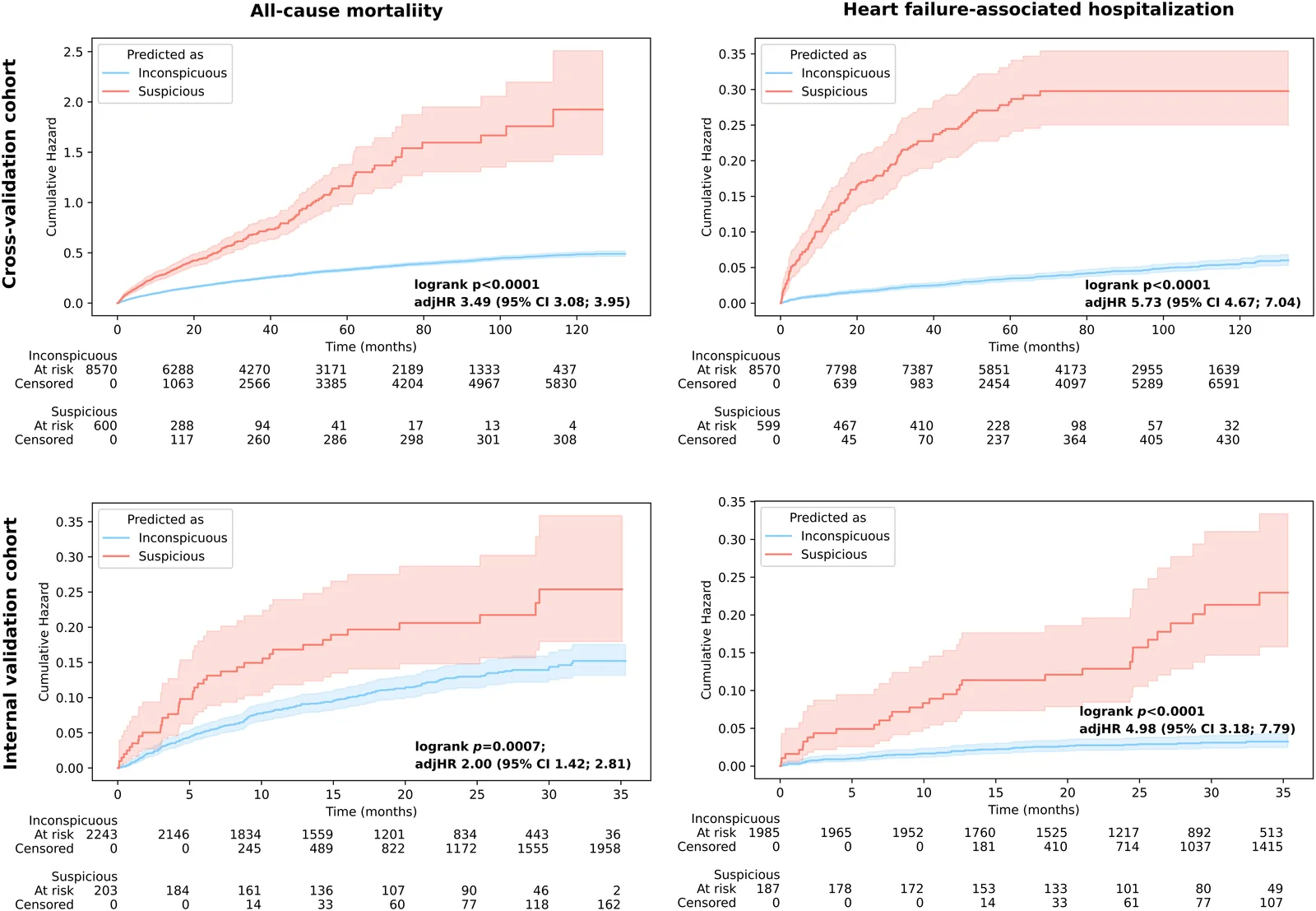
Prognostic value of model predictions. The left column shows Kaplan Meier estimators for all-cause mortality, the right column shows Kaplan Meier estimators for heart failure-associated hospitalization. Adjustment was solely performed for cancer. The upper row shows results for the development (cross-validation) cohort, the lower row shows the results for the independent internal validation.

In addition, separate Cox proportional hazard models were adjusted for cancer, age, sex, BMI, renal insufficiency, atrial fibrillation, hypertension, diabetes, coronary artery disease, COPD, carpal tunnel syndrome, heart-failure history, and interventricular septum thickness. A positive *Amylo-Dete*ct prediction remained independently associated with all-cause mortality (development: adjHR 1.39 [95% CI 1.19; 1.62]; validation: adjHR 1.83 [95% CI 1.19; 2.83]) and HFH (development adjHR 1.39 [95% CI 1.16; 1.67]; validation adjHR 1.50 [95% CI 1.04; 2.16]) (Fig 4).

**Fig 4:**
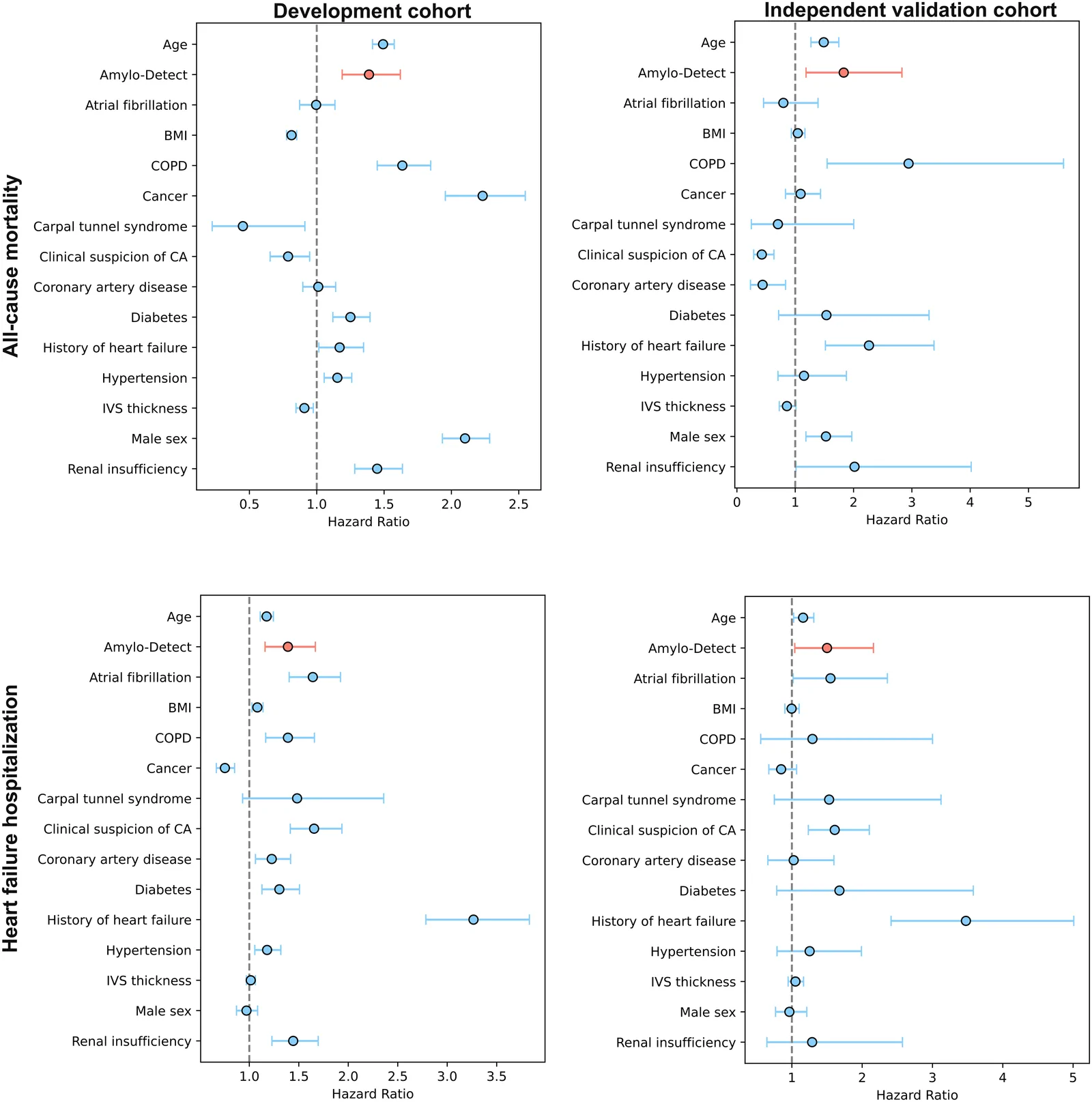
Time-to-event analysis results after multivariate adjustment (cancer, age, sex, BMI, renal insufficiency, atrial fibrillation, hypertension, diabetes, coronary artery disease, COPD, carpal tunnel syndrome, heart-failure history, and interventricular septum thickness) for all-cause mortality and heart failure hospitalization.

## Discussion

In this study, we developed and validated *Amylo-Detect*, a prediction tool for identifying patients at risk for CA based on routinely acquired data from EHRs. *Amylo-Detect* demonstrated excellent performance in predicting high-grade cardiac uptake on subsequent bone scans. Among patients not suspected of having CA at the time of scintigraphy, the model correctly predicted CA-suggestive uptake in 29%, highlighting a potential improvement when used in addition to the current clinical referral pathways. The model also conveyed significant prognostic value for HFH and death. We conclude that *Amylo-Detect* may enable fully automated and reliable identification of at-risk patients for CA, prompting confirmatory testing to enable earlier diagnosis and treatment initiation.

CA represents a systemic disease frequently associated with heterogeneous clinical presentation and multiorgan dysfunction [19]. Extracardiac manifestations, such as carpal tunnel syndrome, may precede CA diagnosis by 5–15 years, thereby creating a window of opportunity for early identification while patients are still free from cardiac symptoms [20]. While the presence of extracardiac indicators individually is non-specific, their cumulative presence may raise suspicion for CA. CA is typically preceded by a prolonged diagnostic interval with multiple healthcare encounters, during which extracardiac features accumulate in the EHRs [21]. These records are exploited by *Amylo-Detect* to raise suspicion among patients with features consistent with a scintigraphy-referral population. Timely treatment initiation is fundamental as novel disease-modifying drugs are available, which yield significant prognostic benefits for affected patients [22]. However, respective drugs are less effective at more advanced disease stages [6,8,23]. We hypothesize that machine learning tools, such as *Amylo-Detect*, may shorten the time to diagnosis and identify CA when modern disease-modifying agents exhibit the highest benefit.

Real-world clinical data, particularly from EHR, inherently contains missing values. Accurate integration of this property is crucial to develop reliable and clinically applicable models. Many published models fail to meet this requirement, necessitating additional examination and therefore reducing acceptance and applicability in clinical routine [9,10,24]. In this study, we handle this issue by applying a machine learning-based imputation approach. We show that this approach yields excellent performance across three large-scale cohorts and several clinically relevant subgroups, demonstrating that *Amylo-Detect* is robust to missingness of individual parameters, including echocardiographic findings.

Our results support three use cases of *Amylo-Detect*. First, risk stratification among patients already referred for scintigraphy. Second, opportunistic identification among patients undergoing scintigraphy for non-cardiac indications. Third, identification of previously missed cases for study cohorts. When employed to improve referral for bone scintigraphy, *Amylo-Detect* is intended to supplement clinical judgment and to prioritize patients for confirmatory bone scintigraphy and monoclonal protein testing. It is not intended to replace clinical assessment. In deployment, it operates alongside routine clinical suspicion and does not override it, so a patient who is clinically suspected of CA is referred regardless of the model output. The model therefore adds rule-in capacity for patients not already flagged by routine and does not remove patients from the existing pathway.

A few studies have demonstrated the potential of machine learning to enhance the detection of CA using merely unimodal approaches in late diagnostic settings [25–27]. Three studies have suggested the possibility of integrating structured imaging and non-imaging data for CA identification and referral [9,10,28]. In related studies, Arana-Achaga et al. focused on the detection among ~1,100 patients with suspected CA while Shiri et al. focused on the detection among ~260 patients with aortic stenosis and Davies et al. predicted presence of CA in a PYP scan referral cohort, a cohort of HFpEF patients, and an additional external PYP scan referral cohort, including a total of ~1000 patients. Our study had a 13- to 50-times larger cohort size, included all-comer referrals for bone scintigraphy, included an external validation on a large-scale cohort, and incorporated prognostic assessment. Unlike the aforementioned studies, this approach enables detection of patients at risk for CA in a real-world setting who would otherwise be missed by mere clinical judgement. *Amylo-Detect* has further demonstrated superior performance with AUCs of 0.93, 0.91, and 0.91 in the development, internal, and external validation cohorts, respectively, compared to AUCs of 0.92 and 0.84 in the study by Arana-Achaga et al. [10], 0.84 in the study by Shiri et al. [28], and AUCs of 0.89, 0.84, and 0.84 for the three cohorts employed by Davies et al. [9]. Additionally, in a head-to-head comparison to the Mayo ATTR-CM score [9] the *Amylo-Detect* model demonstrated substantially improved performance. We further made the developed detection model publicly available via a web-based tool, the *Amylo-Detect* App, to ensure transparency and allow researchers to further validate its use (https://amylo-detect.streamlit.app/). Users can enter the presence or absence of risk factors and are subsequently provided with a predicted probability of CA-suggestive tracer uptake on bone scintigraphy. The web app allows for tabular input of multiple patients, enabling the usage of *Amylo-Detect* for large-scale studies without the necessity of time-consuming single-patient input.

Several strengths and limitations merit comment. As a systemic disease, many factors may contribute to and facilitate detection in CA. Despite our efforts to include a comprehensive set of parameters, some baseline parameters, such as lumbar spinal stenosis, biceps tendon rupture and electrocardiography data [29–31] have not been included due to the reliance on structured data from EHR. *Amylo-Detect* requires structured EHR data to be retrievable at the point of inference. In fragmented systems with limited interoperability between primary care, hospitals and cardiology services, the available data may be sparser than in our integrated setting, and local validation would be required before deployment. Further, while our results suggest that *Amylo-Detect* can facilitate earlier diagnosis by detecting at-risk patients before clinical suspicion is raised, it does not necessarily detect early-stage disease. Notably, while *Amylo-Detect* outperformed existing scores as well as the current clinical standard in most scenarios and metrics, it showed lower sensitivity compared to the clinical standard. Nevertheless, *Amylo-Detect* was able to predict a substantial number of cases not identified in clinical routine. Further, the model provides the possibility to adapt the probability threshold to achieve higher sensitivity at the cost of a reduced positive predictive value. The optimal operating point depends on local resource constraints and the relative cost of a missed diagnosis. A sensitivity-prioritized threshold increases downstream confirmatory imaging but maximizes detection of treatable disease, whereas a balanced threshold reduces scan volume at the cost of some missed cases. Centers can position this threshold according to scanner capacity and budget. In addition, positive predictions by *Amylo-Detect* can be further enhanced by using criteria based on echocardiography and CMR to increase the positive predictive value before patients are sent for final confirmation using scintigraphy and monoclonal protein testing. Development and validation of our model using a broad patient population (all-comer referrals for scintigraphy) ensure real-life applicability. Previous models in the field have been developed using study populations with much higher-than-normal disease prevalence (≥20% vs. 3% in the present cohort), which may yield deceptively good model performance but largely limits their implementation in real-life settings [30,32]. Despite the broad patient population included, it is not feasible to include individuals who did not undergo scintigraphy because of the retrospective study design, as no diagnostic labels would be available for model training. Therefore, our work is subject to a referral bias for bone scintigraphy. We show that *Amylo-Detect* can identify patients at risk of CA-suggestive scintigraphy uptake among patients whose clinical features resemble those of a bone scintigraphy referral population. Although the study population spanned a wide range of clinical indications beyond suspected CA and a wide range of ages and comorbidities, this study was neither developed nor validated in an unselected hospital population. Broader hospital-wide screening requires prospective validation with systematic assessment of CA in all patients. We further performed rigorous external testing and subgroup analysis, yielding high model performance, even for patients with non-cardiac referral indications who would otherwise have been missed by clinical judgement. The publication of the *Amylo-Detect* as web app forms the basis for the prospective evaluation of this concept at multiple independent centers.

In summary, this study presents a novel machine learning-based approach, *Amylo-Detect*, for the accurate identification of patients at risk for CA, integrating a broad spectrum of available EHR data. *Amylo-Detect* exhibited robust diagnostic performance across multiple cohorts and subgroups, achieving high accuracy for the prediction of high-grade cardiac uptake suggestive for CA, and demonstrated significant prognostic value. *Amylo-Detect* identified many patients with “incidental” CA, not suspected by clinical judgement. This highlights its potential to enhance detection and prompt treatment initiation, crucial for improving clinical outcomes in patients with this progressive and fatal disease.

## Supporting information

S1 Table Clinical characteristics of the development cohort. Continuous parameters are given as mean and standard deviation. Binary and ordinal parameters are given as absolute numbers and prevalence. BMI, body mass index; COPD, chronic obstructive pulmonary disease; NT-proBNP, N-terminal pro B-type natriuretic peptide; eGFR, estimated glomerular filtration rate; BUN, blood urea nitrogen; LDL, low-density lipoprotein; HbA1c, Hemoglobin A1c; TSH, thyroid stimulating hormone; LDH, lactate dehydrogenase; CRP, C-reactive protein.
(DOCX)

S2 Table Clinical characteristics of the independent internal validation cohort. Continuous parameters are given as mean and standard deviation. Binary and ordinal parameters are given as absolute numbers and prevalence. BMI, body mass index; COPD, chronic obstructive pulmonary disease; NT-proBNP, N-terminal pro B-type natriuretic peptide; eGFR, estimated glomerular filtration rate; BUN, blood urea nitrogen; LDL, low-density lipoprotein; HbA1c, Hemoglobin A1c; TSH, thyroid stimulating hormone; LDH, lactate dehydrogenase; CRP, C-reactive protein.
(DOCX)

S3 Table Clinical characteristics of the external validation cohort. Continuous parameters are given as mean and standard deviation. Binary and ordinal parameters are given as absolute numbers and prevalence.
(DOCX)

S4 Table Coefficients of the logistic regression model. (DOCX)

S5 Table Performance comparison of Amylo-Detect with existing risk score (Mayo ATTR-CM). Performance of the superior model for each metric and analysis is shown in bold. Better performance in each comparison is displayed in bold. AUC, area under the receiver operating characteristic curve; SNS, sensitivity; SPC, specificity; PPV, positive predictive value; NPV, negative predictive value; NA, not applicable (all patients predicted to be at-risk).
(DOCX)

S1 Fig Referral indications for patients in the development (cross-validation) and independent internal validation data from Vienna. (DOCX)

S2 Fig Cohort flow diagram. (DOCX)

S3 Fig Performance for multiple evaluated algorithms during development in the cross-validation cohort. Area under the receiver operating characteristic curve (AUC) for the logistic regression model which was used for subsequent analyses is shown in blue. Values above the bars indicate AUC values for the corresponding algorithm. LGR, Machine learning-enhanced logistic regression; RF, Random forest; EBM, Explainable boosting machine; XGB, Extreme gradient boosting; SVM, Support vector machine; KNN, k-nearest neighbors; DT, Decision tree; AUC, Area under the curve.
(DOCX)

S4 Fig Calibration of Amylo-Detect for all three cohorts before and after logistic recalibration using the development cohort. Recalibration metrics are provided for the recalibrated model.
(DOCX)

S5 Fig Confusion matrices at threshold 0.043 for the cross-validation development (A), temporal internal validation (B) and external validation (C). (DOCX)

S6 Fig Diagnostic performance for any cardiac uptake (Perugini grade ≥1). (DOCX)

S7 Fig Diagnostic performance at various thresholds. (DOCX)

S8 Fig Decision curve analysis. (DOCX)

S9 Fig Prognostic value of high-grade cardiac uptake in patients not suspected of cardiac amyloidosis at the time of scintigraphy among patients in the two Vienna cohorts. **A)** Heart failure hospitalization. **B)** All-cause mortality. Red Kaplan Meier estimators indicate patients with high-grade cardiac uptake (Perugini grade 2 or 3). Blue Kaplan Meier estimators indicate patients with Perugini grade 0 or 1.
(DOCX)
