# Supplementary material for: Screening for patients at risk for cardiac amyloidosis via electronic health records: A multicenter machine learning development and validation study
Source: PLOS Digit Health. 2026 Oct 8;5(10):e0001637. doi: 10.1371/journal.pdig.0001637 (PMC13649026; doi:10.1371/journal.pdig.0001637)
Supplement: Supplementary file 1 [file pdig.0001637.s006.docx]

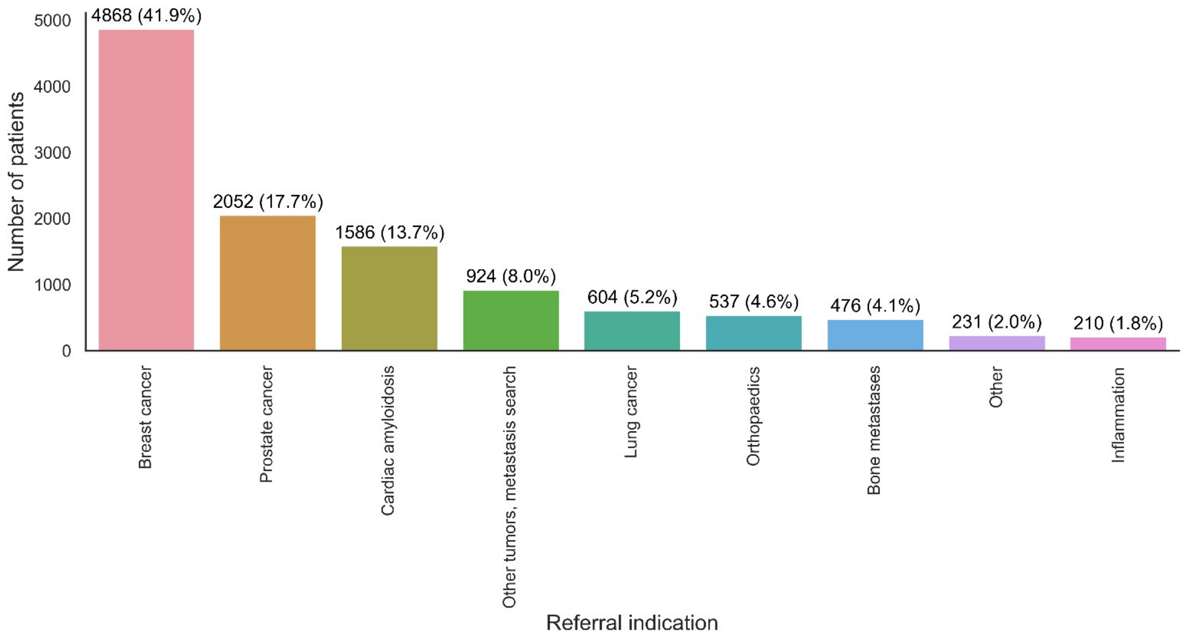
Number of patients
5000
4000
3000
2000
1000
0
4868 (41.9%)
2052 (17.7%)
1586 (13.7%)
924 (8.0%)
604 (5.2%)
537 (4.6%)
476 (4.1%)
231 (2.0%)
210 (1.8%)
Breast cancer
Prostate cancer
Cardiac amyloidosis
Other tumors, metastasis search
Lung cancer
Orthopaedics
Bone metastases
Other
Inflammation
Referral indication
