# Supplementary material for: Screening for patients at risk for cardiac amyloidosis via electronic health records: A multicenter machine learning development and validation study
Source: PLOS Digit Health. 2026 Oct 8;5(10):e0001637. doi: 10.1371/journal.pdig.0001637 (PMC13649026; doi:10.1371/journal.pdig.0001637)
Supplement: Supplementary file 2 [file pdig.0001637.s007.docx]

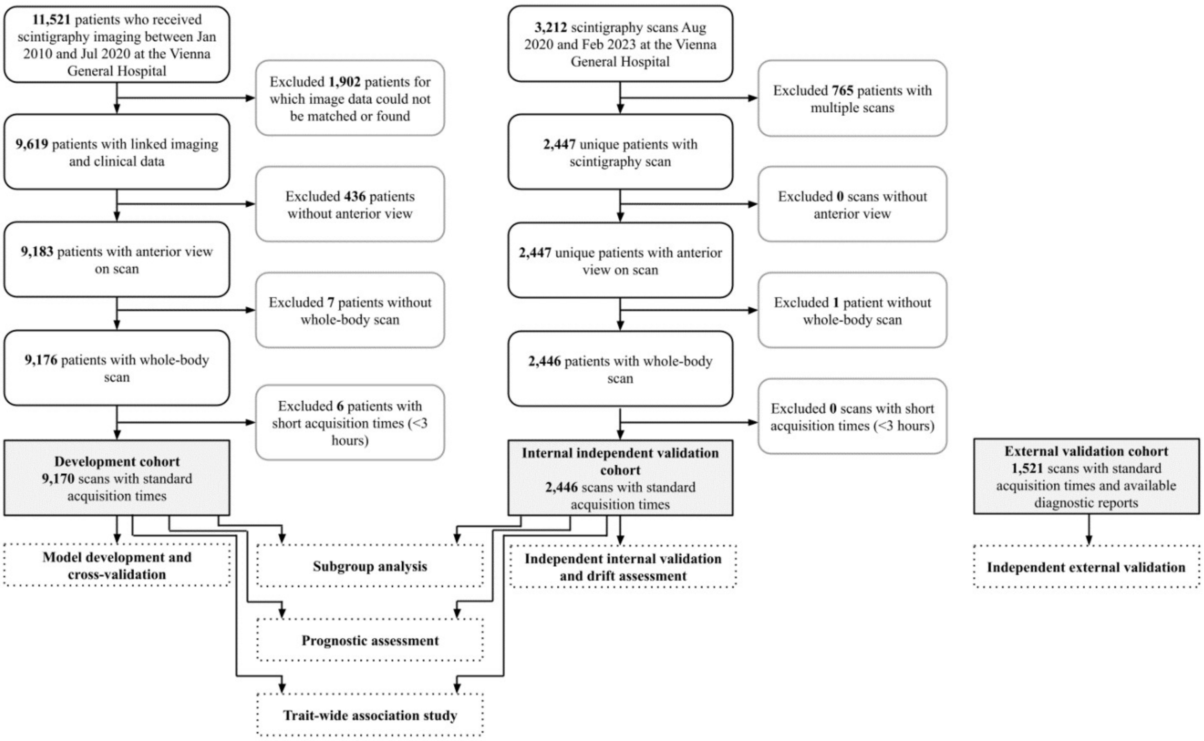
11,521 patients who received scintigraphy imaging between Jan 2010 and Jul 2020 at the Vienna General Hospital
Excluded 1,902 patients for which image data could not be matched or found
9,619 patients with linked imaging and clinical data
Excluded 436 patients without anterior view
9,183 patients with anterior view on scan
Excluded 7 patients without whole-body scan
9,176 patients with whole-body scan
Excluded 6 patients with short acquisition times (<3 hours)
Development cohort 9,170 scans with standard acquisition times
Model development and cross-validation
Subgroup analysis
Prognostic assessment
Trait-wide association study
3,212 scintigraphy scans Aug 2020 and Feb 2023 at the Vienna General Hospital
Excluded 765 patients with multiple scans
2,447 unique patients with scintigraphy scan
Excluded 0 scans without anterior view
2,447 unique patients with anterior view on scan
Excluded 1 patient without whole-body scan
2,446 patients with whole-body scan
Excluded 0 scans with short acquisition times (<3 hours)
Internal independent validation cohort 2,446 scans with standard acquisition times
Independent internal validation and drift assessment
External validation cohort 1,521 scans with standard acquisition times and available diagnostic reports
Independent external validation
