# Supplementary material for: Screening for patients at risk for cardiac amyloidosis via electronic health records: A multicenter machine learning development and validation study
Source: PLOS Digit Health. 2026 Oct 8;5(10):e0001637. doi: 10.1371/journal.pdig.0001637 (PMC13649026; doi:10.1371/journal.pdig.0001637)
Supplement: Supplementary file 3 [file pdig.0001637.s008.docx]

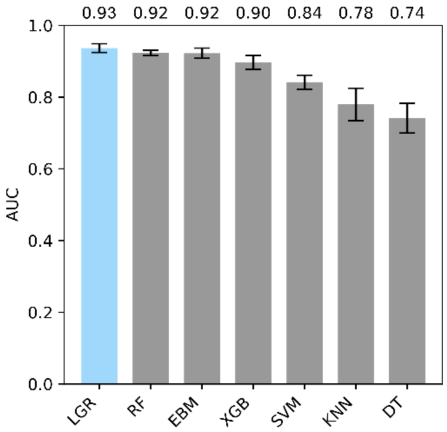
0.93
0.92
0.92
0.90
0.84
0.78
0.74
AUC
1.0
0.8
0.6
0.4
0.2
0.0
LGR
RF
EBM
XGB
SVM
KNN
DT
