# Supplementary material for: Screening for patients at risk for cardiac amyloidosis via electronic health records: A multicenter machine learning development and validation study
Source: PLOS Digit Health. 2026 Oct 8;5(10):e0001637. doi: 10.1371/journal.pdig.0001637 (PMC13649026; doi:10.1371/journal.pdig.0001637)
Supplement: Supplementary file 4 [file pdig.0001637.s009.docx]

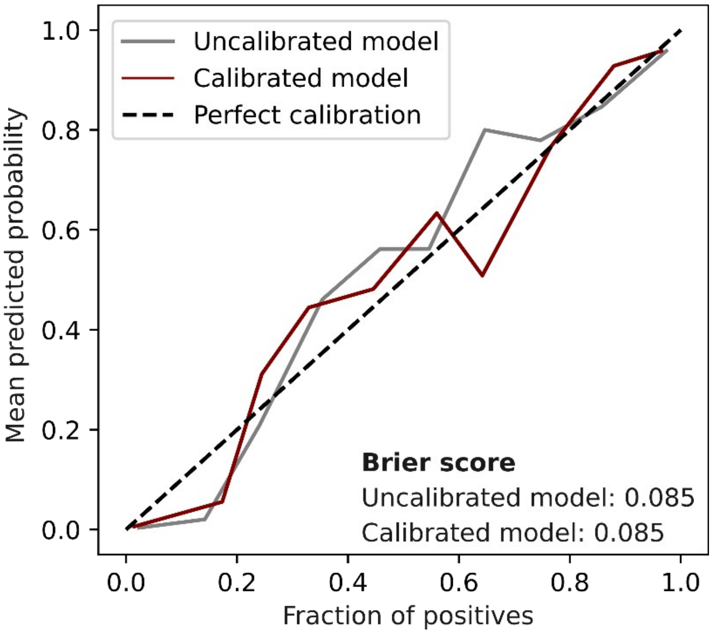
Uncalibrated model
Calibrated model
Perfect calibration
Mean predicted probability
Fraction of positives
0.0
0.2
0.4
0.6
0.8
1.0
Brier score
Uncalibrated model: 0.085
Calibrated model: 0.085
