# Supplementary material for: Screening for patients at risk for cardiac amyloidosis via electronic health records: A multicenter machine learning development and validation study
Source: PLOS Digit Health. 2026 Oct 8;5(10):e0001637. doi: 10.1371/journal.pdig.0001637 (PMC13649026; doi:10.1371/journal.pdig.0001637)
Supplement: Supplementary file 5 [file pdig.0001637.s010.docx]

A

		Predicted	
		Grade ≤1	Grade ≥2
True	Grade 1	8387	641
	True grade 2	41	101

B

		Predicted	
		Grade ≤1	Grade ≥2
True	Grade 1	2207	138
	True grade 2	36	65

C

		Predicted	
		Grade ≤1	Grade ≥2
True	Grade 1	1213	163
	True grade 2	39	106
