# Supplementary material for: Screening for patients at risk for cardiac amyloidosis via electronic health records: A multicenter machine learning development and validation study
Source: PLOS Digit Health. 2026 Oct 8;5(10):e0001637. doi: 10.1371/journal.pdig.0001637 (PMC13649026; doi:10.1371/journal.pdig.0001637)
Supplement: Supplementary file 6 [file pdig.0001637.s011.docx]

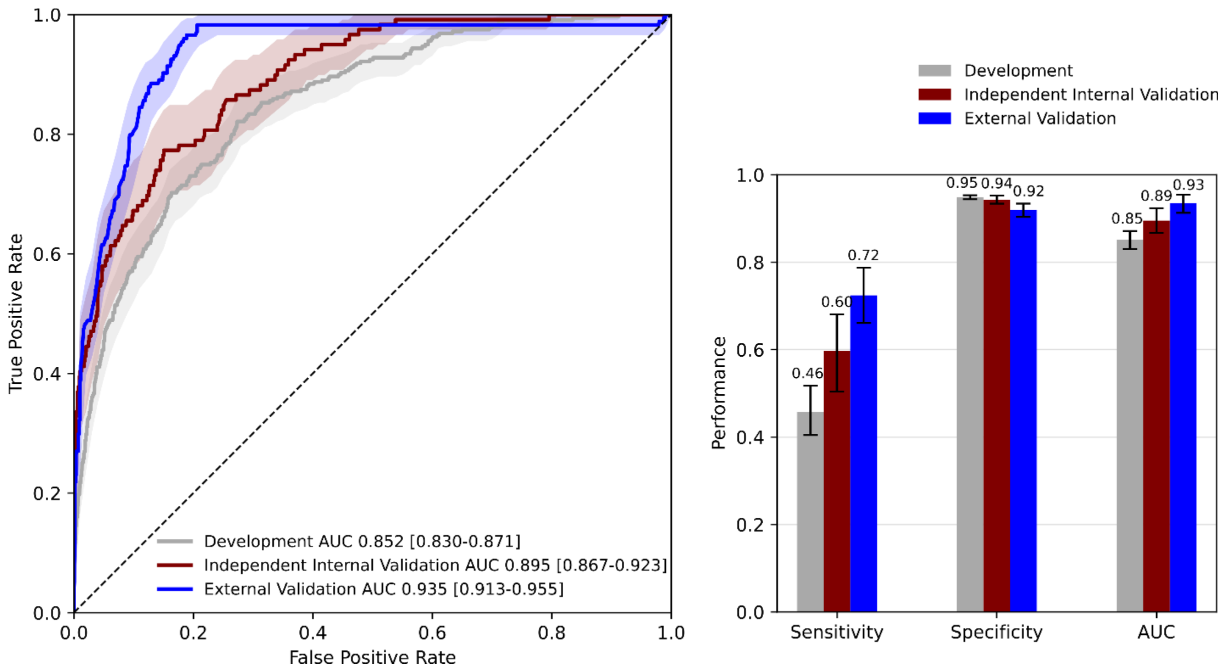

True Positive Rate
False Positive Rate
Development AUC 0.852 [0.830-0.871]
Independent Internal Validation AUC 0.895 [0.867-0.923]
External Validation AUC 0.935 [0.913-0.955]
Development
Independent Internal Validation
External Validation
Performance
Sensitivity
Specificity
AUC
0.46
0.60
0.72
0.95
0.94
0.92
0.85
0.89
0.93
