# Supplementary material for: Screening for patients at risk for cardiac amyloidosis via electronic health records: A multicenter machine learning development and validation study
Source: PLOS Digit Health. 2026 Oct 8;5(10):e0001637. doi: 10.1371/journal.pdig.0001637 (PMC13649026; doi:10.1371/journal.pdig.0001637)
Supplement: Supplementary file 7 [file pdig.0001637.s012.docx]

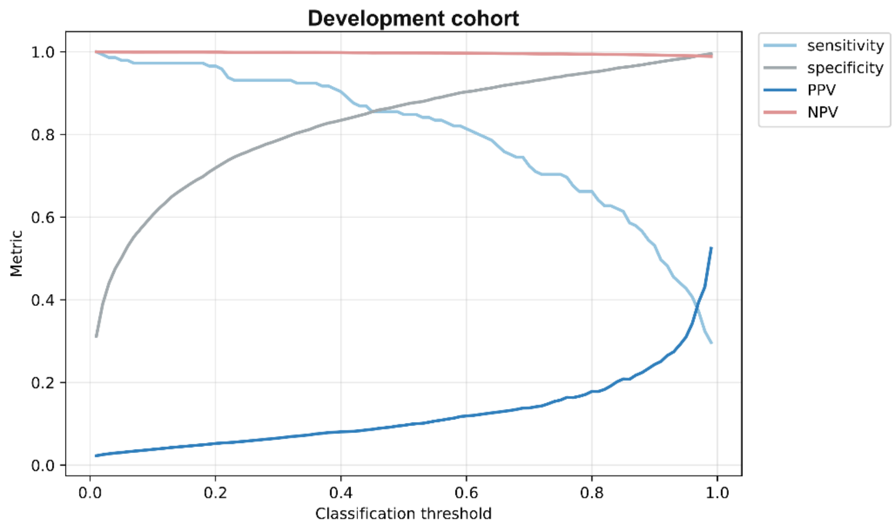
Development cohort
sensitivity
specificity
PPV
NPV
Metric
Classification threshold

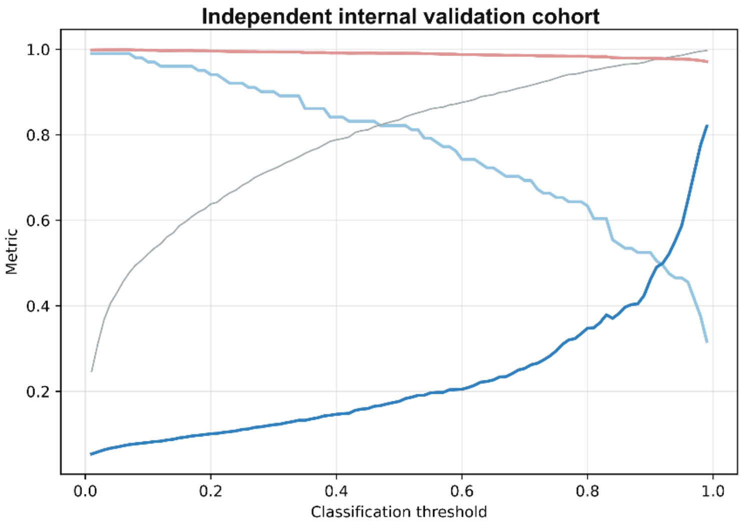
Independent internal validation cohort
Metric
Classification threshold

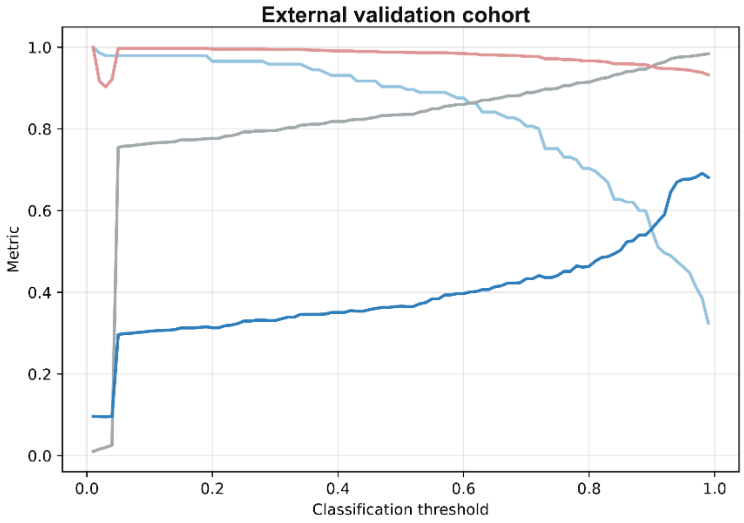
External validation cohort
Metric
Classification threshold
