# Supplementary material for: Screening for patients at risk for cardiac amyloidosis via electronic health records: A multicenter machine learning development and validation study
Source: PLOS Digit Health. 2026 Oct 8;5(10):e0001637. doi: 10.1371/journal.pdig.0001637 (PMC13649026; doi:10.1371/journal.pdig.0001637)
Supplement: Supplementary file 8 [file pdig.0001637.s013.docx]

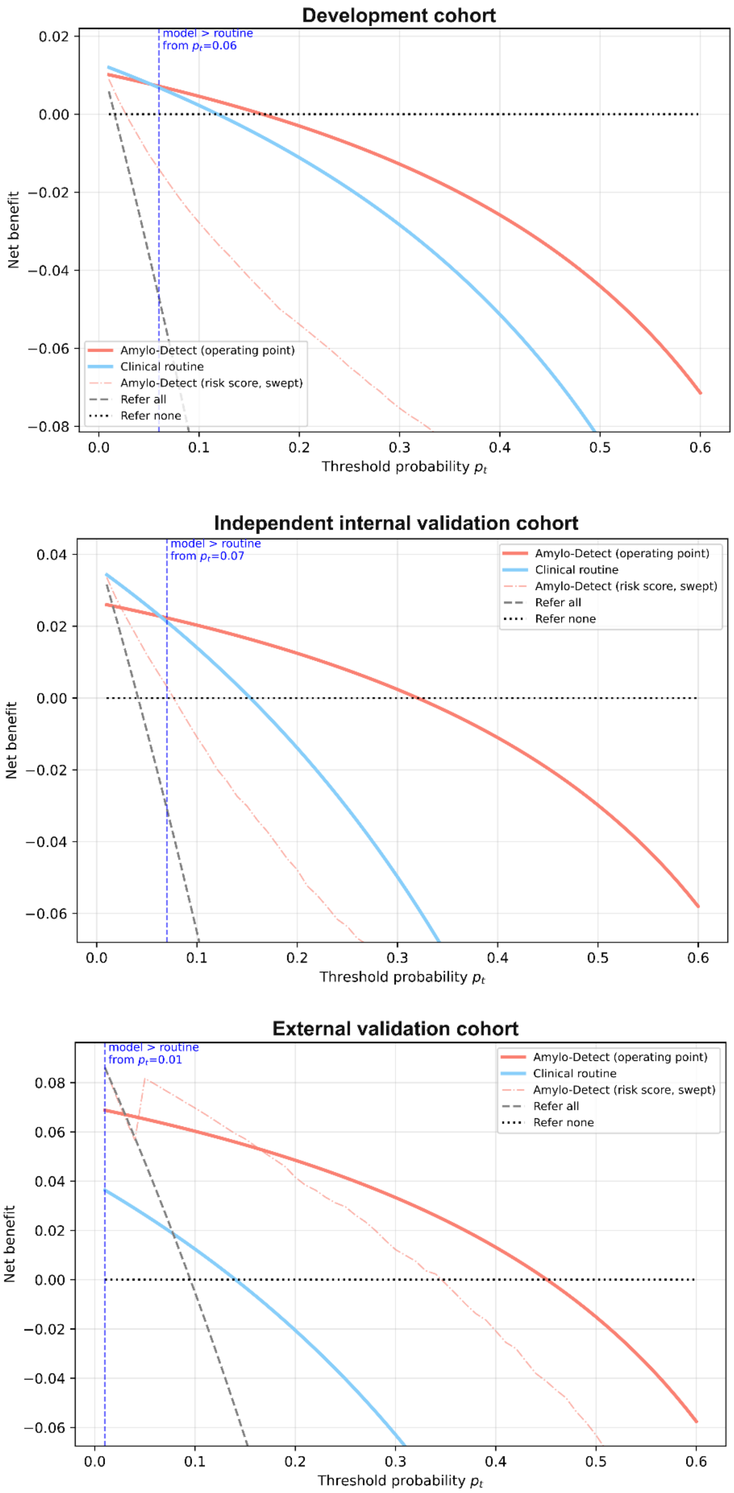

Development cohort
model > routine
from p_t=0.06
Net benefit
Threshold probability p_t
Amylo-Detect (operating point)
Clinical routine
Amylo-Detect (risk score, swept)
Refer all
Refer none
Independent internal validation cohort
model > routine
from p_t=0.07
Net benefit
Threshold probability p_t
Amylo-Detect (operating point)
Clinical routine
Amylo-Detect (risk score, swept)
Refer all
Refer none
External validation cohort
model > routine
from p_t=0.01
Net benefit
Threshold probability p_t
Amylo-Detect (operating point)
Clinical routine
Amylo-Detect (risk score, swept)
Refer all
Refer none
