# Supplementary material for: Screening for patients at risk for cardiac amyloidosis via electronic health records: A multicenter machine learning development and validation study
Source: PLOS Digit Health. 2026 Oct 8;5(10):e0001637. doi: 10.1371/journal.pdig.0001637 (PMC13649026; doi:10.1371/journal.pdig.0001637)
Supplement: Supplementary file 9 [file pdig.0001637.s014.docx]

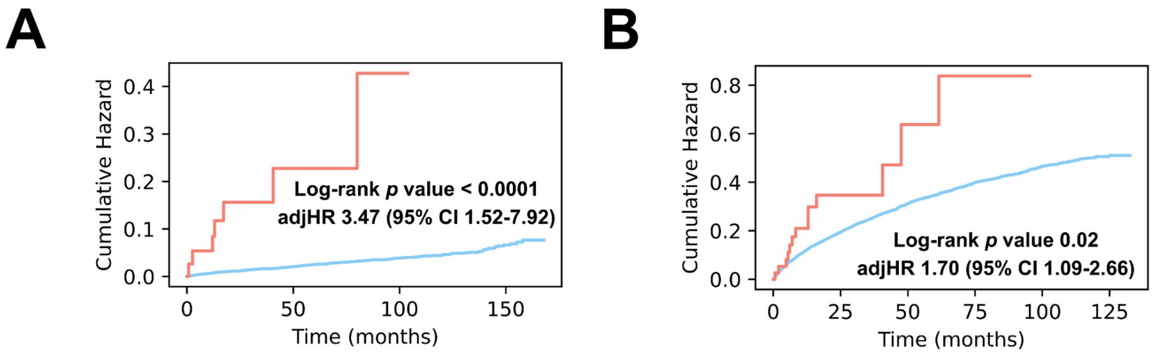


**A**

| | | | | |
|---|---|---|---|---|
| No cardiac uptake | | | | |
| At risk | 9798 | 6448 | 2993 | 460 |
| Censored | 0 | 3173 | 6542 | 9042 |
| High cardiac uptake | | | | |
| At risk | 38 | 10 | 2 | 0 |
| Censored | 0 | 22 | 29 | 31 |

**B**

| | | | | | | |
|---|---|---|---|---|---|---|
| No cardiac uptake | | | | | | |
| At risk | 9992 | 6247 | 3757 | 2397 | 1346 | 214 |
| Censored | 0 | 2091 | 4026 | 5123 | 6048 | 7143 |
| High cardiac uptake | | | | | | |
| At risk | 38 | 17 | 5 | 1 | 0 | 0 |
| Censored | 0 | 11 | 21 | 24 | 25 | 25 |
