# Supplementary material for: Screening for patients at risk for cardiac amyloidosis via electronic health records: A multicenter machine learning development and validation study
Source: PLOS Digit Health. 2026 Oct 8;5(10):e0001637. doi: 10.1371/journal.pdig.0001637 (PMC13649026; doi:10.1371/journal.pdig.0001637)
Supplement: Supplementary file 1 [file pdig.0001637.s001.docx]

| Parameter | Parameter value | *p* value | Entire data | No high-grade uptake | High-grade uptake |
| --- | --- | --- | --- | --- | --- |
| Number of patients, n (%) |  |  | 9,170 (100.00%) | 9,028 (98.45%) | 142 (1.55%) |
| High-grade cardiac uptake (Perugini grade 2 or 3) |  | < 0.0001 | 142 (1.55%) | 0 (0%) | 142 (100.0%) |
| Age (years) |  | < 0.0001 | 61.77 (15.73) | 61.5 (15.67) | 78.68 (8.41) |
| BMI (kg/m^2^) |  | 0.2606 | 26.38 (5.32) | 26.39 (5.33) | 25.71 (4.27) |
|  | NA |  | 277 (3.02%) | 274 (3.04%) | 3 (2.11%) |
| Male sex |  | < 0.0001 | 3,326 (36.27%) | 3,218 (35.64%) | 108 (76.05%) |
| **Comorbidities** | | | | | |
| Atrial fibrillation |  | < 0.0001 | 857 (9.35%) | 786 (8.71%) | 71 (50.0%) |
| History of any bleeding event |  | 0.8483 | 481 (5.25%) | 473 (5.24%) | 8 (5.63%) |
| History of cerebral bleeding |  | 0.3858 | 86 (0.94%) | 84 (0.93%) | 2 (1.41%) |
| Carpal tunnel syndrome |  | < 0.0001 | 62 (0.68%) | 34 (0.38%) | 28 (19.72%) |
| COPD |  | 0.1363 | 657 (7.16%) | 642 (7.11%) | 15 (10.56%) |
| Coronary artery disease |  | < 0.0001 | 1,201 (13.1%) | 1,162 (12.87%) | 39 (27.46%) |
| History of myocardial infarction |  | 0.1973 | 174 (1.9%) | 169 (1.87%) | 5 (3.52%) |
| Diabetes |  | 0.7973 | 1,131 (12.33%) | 1,115 (12.35%) | 16 (11.27%) |
| History of heart failure |  | < 0.0001 | 828 (9.03%) | 767 (8.5%) | 61 (42.96%) |
| Hyperlipidemia |  | 0.5967 | 250 (2.73%) | 248 (2.75%) | 2 (1.41%) |
| Hypertension |  | 0.1209 | 3,723 (40.6%) | 3,656 (40.5%) | 67 (47.18%) |
| Liver disease |  | 0.0227 | 418 (4.56%) | 417 (4.62%) | 1 (0.7%) |
| Myeloma |  | 1.0000 | 93 (1.01%) | 92 (1.02%) | 1 (0.7%) |
| Renal insufficiency (ICD-10) |  | < 0.0001 | 784 (8.55%) | 755 (8.36%) | 29 (20.42%) |
| History of stroke |  | 0.0382 | 507 (5.53%) | 493 (5.46%) | 14 (9.86%) |
| **Blood parameters** | | | | | |
| NT-proBNP (pg/mL) |  | < 0.0001 | 2,848 (5,292) | 2,700 (5,220) | 5,227 (6,123) |
|  | NA |  | 7,488 (81.66%) | 7,450 (82.52%) | 38 (26.76%) |
| Creatinine (µmol/L) |  | < 0.0001 | 1.05 (0.8) | 1.04 (0.8) | 1.34 (0.77) |
|  | NA |  | 4,034 (43.99%) | 4,012 (44.44%) | 22 (15.49%) |
| eGFR (mL/min/1.73 m²) |  | < 0.0001 | 80.14 (31.12) | 80.57 (31.17) | 62.13 (22.55) |
|  | NA |  | 4,034 (43.99%) | 4,012 (44.44%) | 22 (15.49%) |
| BUN (mg/dL) |  | < 0.0001 | 17.84 (10.39) | 17.6 (10.09) | 27.67 (16.09) |
|  | NA |  | 4,090 (44.60%) | 4,068 (45.06%) | 22 (15.49%) |
| Triglycerides (mg/dL) |  | < 0.0001 | 131.15 (74.61) | 132.21 (75.01) | 90.05 (38.8) |
|  | NA |  | 5,376 (58.63%) | 5,330 (59.04%) | 46 (32.39%) |
| Cholesterol (mg/dL) |  | < 0.0001 | 183.12 (52.01) | 183.81 (52.01) | 157.01 (44.74) |
|  | NA |  | 5,422 (59.13%) | 5,376 (59.55%) | 46 (32.39%) |
| LDL (mg/dL) |  | < 0.0001 | 105.08 (43.35) | 105.86 (43.36) | 82.27 (36.21) |
|  | NA |  | 6,995 (76.28%) | 6,925 (76.71%) | 70 (49.30%) |
| Bilirubin (mg/dL) |  | < 0.0001 | 0.68 (1.13) | 0.68 (1.14) | 0.85 (0.58) |
|  | NA |  | 4,488 (48.94%) | 4,461 (49.41%) | 27 (19.01%) |
| Hemoglobin (g/dL) |  | 0.0348 | 12.61 (1.93) | 12.6 (1.94) | 12.98 (1.72) |
|  | NA |  | 4,209 (45.90%) | 4,190 (46.41%) | 19 (13.38%) |
| Hematocrit (%) |  | 0.0199 | 37.46 (5.41) | 37.43 (5.42) | 38.61 (4.85) |
|  | NA |  | 4,209 (45.90%) | 4,190 (46.41%) | 19 (13.38%) |
| HbA1c (%) |  | 0.0583 | 5.92 (1.11) | 5.92 (1.13) | 5.95 (0.73) |
|  | NA |  | 7,529 (82.10%) | 7,450 (82.52%) | 79 (55.63%) |
| TSH (mIU/L) |  | 0.8108 | 2.16 (3.02) | 2.14 (2.9) | 2.64 (5.74) |
|  | NA |  | 6,319 (68.91%) | 6,257 (69.31%) | 62 (43.66%) |
| LDH (U/L) |  | < 0.0001 | 230.02 (178.64) | 229.64 (179.89) | 246.61 (111.07) |
|  | NA |  | 4,607 (50.24%) | 4,569 (50.61%) | 38 (26.76%) |
| Troponin (ng/L) |  | < 0.0001 | 101.82 (507.68) | 101.01 (519.86) | 116.17 (192.15) |
|  | NA |  | 8,164 (89.03%) | 8,076 (89.46%) | 88 (61.97%) |
| Creatine kinase (U/L) |  | 0.0502 | 178.83 (436.13) | 179.41 (440.94) | 158.65 (203.31) |
|  | NA |  | 5,806 (63.32%) | 5,757 (63.77%) | 49 (34.51%) |
| Creatine kinase MB (U/L) |  | 0.7851 | 69.31 (164.79) | 70.37 (167.22) | 41.16 (69.1) |
|  | NA |  | 8,645 (94.27%) | 8,522 (94.40%) | 123 (86.62%) |
| CRP (mg/L) |  | 0.0179 | 3.45 (6.27) | 3.47 (6.28) | 2.44 (5.76) |
|  | NA |  | 4,389 (47.86%) | 4,360 (48.29%) | 29 (20.42%) |
| **Echocardiography** | | | | | |
| Left ventricular end-diastolic diameter (mm) |  | 0.0024 | 44.81 (16.37) | 44.94 (16.61) | 41.33 (6.12) |
|  | NA |  | 7,879 (85.92%) | 7,782 (86.20%) | 97 (68.31%) |
| Left atrial diameter (mm) |  | < 0.0001 | 54.13 (9.18) | 53.82 (9.07) | 62.39 (8.27) |
|  | NA |  | 7,939 (86.58%) | 7,841 (86.85%) | 98 (69.01%) |
| Right ventricular end-diastolic diameter (mm) |  | 0.0817 | 31.9 (8.2) | 31.84 (8.26) | 33.57 (6.24) |
|  | NA |  | 7,888 (86.02%) | 7,792 (86.31%) | 96 (67.61%) |
| Right atrial diameter (mm) |  | < 0.0001 | 52.61 (8.64) | 52.31 (8.5) | 60.47 (8.46) |
|  | NA |  | 7,894 (86.09%) | 7,799 (86.39%) | 95 (66.90%) |
| IVS thickness (mm) |  | < 0.0001 | 14.14 (5.19) | 13.9 (5.08) | 20.07 (4.29) |
|  | NA |  | 8,012 (87.37%) | 7,914 (87.66%) | 98 (69.01%) |
| Tricuspid regurgitation | < moderate | < 0.0001 | 1,749 (19.07%) | 1,704 (18.88%) | 45 (31.03%) |
|  | ≥ moderate |  | 215 (2.34%) | 189 (2.09%) | 26 (17.93%) |
|  | NA |  | 7,206 (78.58%) | 7,132 (79.02%) | 74 (51.03%) |
| Pulmonary regurgitation | < moderate | 1.0000 | 1,957 (21.34%) | 1,886 (20.9%) | 71 (48.97%) |
|  | ≥ moderate |  | 7 (0.08%) | 7 (0.08%) | 0 (0%) |
|  | NA |  | 7,206 (78.58%) | 7,132 (79.02%) | 74 (51.03%) |
| Mitral regurgitation | < moderate | < 0.0001 | 1,782 (19.43%) | 1,731 (19.18%) | 51 (35.17%) |
|  | ≥ moderate |  | 182 (1.98%) | 162 (1.8%) | 20 (13.79%) |
|  | NA |  | 7,206 (78.58%) | 7,132 (79.02%) | 74 (51.03%) |
| Mitral stenosis | < moderate | 1.0000 | 1,962 (21.4%) | 1,891 (20.95%) | 71 (48.97%) |
|  | ≥ moderate |  | 2 (0.02%) | 2 (0.02%) | 0 (0%) |
|  | NA |  | 7,206 (78.58%) | 7,132 (79.02%) | 74 (51.03%) |
| Aortic regurgitation | < moderate | 0.5600 | 1,927 (21.01%) | 1,857 (20.58%) | 70 (48.28%) |
|  | ≥ moderate |  | 22 (0.24%) | 21 (0.23%) | 1 (0.69%) |
|  | NA |  | 7,221 (78.75%) | 7,147 (79.19%) | 74 (51.03%) |
| Aortic stenosis | < moderate | 0.6395 | 1,822 (19.87%) | 1,757 (19.47%) | 65 (44.83%) |
|  | ≥ moderate |  | 142 (1.55%) | 136 (1.51%) | 6 (4.14%) |
|  | NA |  | 7,206 (78.58%) | 7,132 (79.02%) | 74 (51.03%) |
| Left ventricular function (diastolic) | Normal | < 0.0001 | 131 (1.43%) | 131 (1.45%) | 0 (0%) |
|  | Grade 1 dysfunction |  | 584 (6.37%) | 578 (6.4%) | 6 (4.23%) |
|  | Grade 2 dysfunction |  | 115 (1.25%) | 112 (1.24%) | 3 (2.11%) |
|  | Grade 3 dysfunction |  | 13 (0.14%) | 8 (0.09%) | 5 (3.52%) |
|  | Grade 4 dysfunction |  | 48 (0.52%) | 39 (0.43%) | 9 (6.34%) |
|  | NA |  | 8,279 (90.28%) | 8,160 (90.39%) | 119 (83.8%) |
| Left ventricular function (systolic) | Preserved | < 0.0001 | 1,072 (11.69%) | 1,051 (11.64%) | 21 (14.79%) |
|  | Mildly reduced |  | 110 (1.52%) | 128 (1.42%) | 12 (8.45%) |
|  | Moderately reduced |  | 59 (0.65%) | 54 (0.6%) | 5 (3.52%) |
|  | Severely reduced |  | 68 (0.74%) | 51 (0.68%) | 7 (4.93%) |
|  | NA |  | 7,831 (85.40%) | 7,734 (85.67%) | 97 (68.31%) |
| **Cardiac MRI** | | | | | |
| Left ventricular ejection fraction (%) |  | 0.0765 | 56.57 (15.66) | 57.1 (15.65) | 51.51 (14.83) |
|  | NA |  | 8,948 (97.58%) | 8,827 (97.77%) | 121 (85.21%) |
| Right ventricular ejection fraction (%) |  | 0.0739 | 51.76 (12.32) | 52.21 (12.18) | 47.5 (12.8) |
|  | NA |  | 8,949 (97.59%) | 8,828 (97.78%) | 121 (85.21%) |
| Right ventricular end-diastolic diameter (mm) (mm) |  | 0.8670 | 39.61 (7.27) | 39.54 (6.99) | 40.24 (9.4) |
|  | NA |  | 8,954 (97.64%) | 8,833 (97.84%) | 121 (85.21%) |
| Left ventricular end-diastolic volume (mL) |  | 0.1473 | 155.76 (53.62) | 154.04 (52.96) | 172.1 (57.01) |
|  | NA |  | 8,950 (97.60%) | 8,829 (97.80%) | 121 (85.21%) |
| Right ventricular end-diastolic volume (mL) |  | 0.0401 | 152.19 (51.08) | 149.75 (50.01) | 175.24 (55.23) |
|  | NA |  | 8,950 (97.60%) | 8,829 (97.80%) | 121 (85.21%) |
| Left ventricular end-systolic volume (mL) |  | 0.0435 | 71.65 (45.55) | 69.74 (44.59) | 89.81 (50.25) |
|  | NA |  | 8,950 (97.60%) | 8,829 (97.80%) | 121 (85.21%) |
| Left ventricular stroke volume (mL) |  | 0.9684 | 84.18 (26.56) | 84.37 (27.07) | 82.38 (21.1) |
|  | NA |  | 8,950 (97.60%) | 8,829 (97.80%) | 121 (85.21%) |
| Right ventricular stroke volume (mL) |  | 0.5604 | 76.88 (25.59) | 76.68 (26.08) | 78.81 (20.3) |
|  | NA |  | 8,950 (97.60%) | 8,829 (97.80%) | 121 (85.21%) |
| Left ventricular cardiac output (L/min) |  | 0.6797 | 5.52 (1.66) | 5.53 (1.71) | 5.51 (1.03) |
|  | NA |  | 8,950 (97.60%) | 8,829 (97.80%) | 121 (85.21%) |
| Right ventricular cardiac output (L/min) |  | 0.1508 | 5.05 (1.65) | 5.02 (1.7) | 5.3 (1.14) |
|  | NA |  | 8,950 (97.60%) | 8,829 (97.80%) | 121 (85.21%) |
| Left ventricular cardiac index (L/min/m²) |  | 0.4452 | 2.95 (0.93) | 2.94 (0.96) | 3.02 (0.52) |
|  | NA |  | 9,008 (98.23%) | 8,880 (98.36%) | 128 (90.14%) |
| Right ventricular cardiac index (L/min/m²) |  | 0.1373 | 2.67 (0.87) | 2.65 (0.89) | 2.9 (0.59) |
|  | NA |  | 9,004 (98.19%) | 8,876 (98.32%) | 128 (90.14%) |
| Left ventricular end-systolic diameter (mm) |  | 0.5331 | 47.35 (7.29) | 47.45 (7.11) | 46.45 (8.68) |
|  | NA |  | 8,951 (97.61%) | 8,831 (97.82%) | 120 (84.51%) |
| Left ventricular end-diastolic diameter (mm) |  | 0.5331 | 47.35 (7.29) | 47.45 (7.11) | 46.45 (8.68) |
|  | NA |  | 8,951 (97.61%) | 8,831 (97.82%) | 120 (84.51%) |
| Extracellular volume (%) |  | < 0.0001 | 30.05 (8.61) | 28.83 (6.99) | 43.78 (12.33) |
|  | NA |  | 8,987 (98.00%) | 8,860 (98.14%) | 127 (89.44%) |
