# Supplementary material for: Screening for patients at risk for cardiac amyloidosis via electronic health records: A multicenter machine learning development and validation study
Source: PLOS Digit Health. 2026 Oct 8;5(10):e0001637. doi: 10.1371/journal.pdig.0001637 (PMC13649026; doi:10.1371/journal.pdig.0001637)
Supplement: Supplementary file 2 [file pdig.0001637.s002.docx]

| Parameter | Parameter value | *p* value | Entire data | No high-grade uptake | High-grade uptake |
| --- | --- | --- | --- | --- | --- |
| Number of patients, n (%) |  |  | 2,446 (100.00%) | 2,345 (95.87%) | 101 (4.13%) |
| High-grade cardiac uptake (Perugini grade 2 or 3) |  | < 0.0001 | 101 (4.13%) | 0 (0%) | 101 (100.0%) |
| Age (years) |  | < 0.0001 | 64.08 (15.91) | 63.43 (15.84) | 79.2 (8.13) |
| BMI (kg/m^2^) |  | 0.4362 | 27.12 (5.19) | 27.07 (5.17) | 28.96 (5.36) |
|  | NA |  | 2,201 (89.98%) | 2,106 (89.81%) | 95 (94.06%) |
| Male sex |  | < 0.0001 | 1,325 (54.17%) | 1,242 (52.96%) | 83 (82.18%) |
| **Comorbidities** | | | | | |
| Atrial fibrillation |  | 0.0004 | 124 (5.07%) | 110 (4.69%) | 14 (13.86%) |
| History of any bleeding event |  | 0.7209 | 49 (2.0%) | 48 (2.05%) | 1 (0.99%) |
| History of cerebral bleeding |  | < 0.0001 | 35 (1.43%) | 10 (0.43%) | 25 (24.75%) |
| Carpal tunnel syndrome |  | 0.4476 | 48 (1.96%) | 45 (1.92%) | 3 (2.97%) |
| COPD |  | 1 | 34 (1.39%) | 33 (1.41%) | 1 (0.99%) |
| Coronary artery disease |  | 0.0277 | 118 (4.82%) | 108 (4.61%) | 10 (9.9%) |
| History of myocardial infarction |  | 1 | 39 (1.59%) | 38 (1.62%) | 1 (0.99%) |
| Diabetes |  | < 0.0001 | 145 (5.93%) | 126 (5.37%) | 19 (18.81%) |
| History of heart failure |  | 0.2919 | 26 (1.06%) | 24 (1.02%) | 2 (1.98%) |
| Hyperlipidemia |  | 0.4633 | 112 (4.58%) | 106 (4.52%) | 6 (5.94%) |
| Hypertension |  | 0.6229 | 25 (1.02%) | 25 (1.07%) | 0 (0%) |
| Liver disease | NA |  | 2,446 (100.0%) | 2,345 (100.0%) | 101 (100.0%) |
| Myeloma |  | 0.6640 | 37 (1.51%) | 35 (1.49%) | 2 (1.98%) |
| Renal insufficiency (ICD-10) |  | 0.0456 | 48 (1.96%) | 43 (1.83%) | 5 (4.95%) |
| History of stroke |  | 0.0366 | 31 (1.27%) | 27 (1.15%) | 4 (3.96%) |
| **Blood parameters** | | | | | |
| NT-proBNP (pg/mL) |  | < 0.0001 | 2,066 (5,322) | 1,873 (5,217) | 4,481 (5,991) |
|  | NA |  | 1,378 (56.34%) | 1,356 (57.83%) | 22 (21.78%) |
| Creatinine (µmol/L) |  | < 0.0001 | 1.05 (0.82) | 1.04 (0.82) | 1.36 (0.65) |
|  | NA |  | 580 (23.71%) | 569 (24.26%) | 11 (10.89%) |
| eGFR (mL/min/1.73 m²) | NA |  | 2,446 (100.00%) | 2,345 (100.00%) | 101 (100.00%) |
|  |  | < 0.0001 | 18.43 (10.8) | 17.9 (10.05) | 28.86 (17.55) |
| BUN (mg/dL) | NA |  | 595 (24.33%) | 584 (24.90%) | 11 (10.89%) |
|  |  | < 0.0001 | 133.37 (82.91) | 135.22 (84.11) | 97.25 (39.9) |
| Triglycerides (mg/dL) | NA |  | 891 (36.43%) | 866 (36.93%) | 25 (24.75%) |
|  |  | < 0.0001 | 175.82 (53.37) | 177.28 (53.3) | 147.45 (46.35) |
| Cholesterol (mg/dL) | NA |  | 929 (37.98%) | 902 (38.46%) | 27 (26.73%) |
|  |  |  |  |  |  |
| LDL (mg/dL) | NA |  | 2,446 (100.00%) | 2,345 (100.00%) | 101 (100.00%) |
|  |  | < 0.0001 | 0.56 (0.52) | 0.54 (0.51) | 0.85 (0.62) |
| Bilirubin (mg/dL) | NA |  | 642 (26.25%) | 631 (26.91%) | 11 (10.89%) |
|  |  | 0.2326 | 12.92 (1.92) | 12.91 (1.92) | 13.19 (2.05) |
| Hemoglobin (g/dL) | NA |  | 604 (24.69%) | 593 (25.29%) | 11 (10.89%) |
|  |  | 0.1003 | 38.67 (5.35) | 38.62 (5.34) | 39.61 (5.63) |
| Hematocrit (%) | NA |  | 604 (24.69%) | 593 (25.29%) | 11 (10.89%) |
|  |  | 0.0176 | 5.95 (1.11) | 5.95 (1.14) | 5.95 (0.72) |
| HbA1c (%) | NA |  | 1,496 (61.16%) | 1,459 (62.22%) | 37 (36.63%) |
|  |  | 0.4724 | 2.08 (2.46) | 2.09 (2.51) | 1.84 (1.27) |
| TSH (mIU/L) | NA |  | 1,191 (48.69%) | 1,156 (49.30%) | 35 (34.65%) |
|  |  | < 0.0001 | 223.21 (121.62) | 222.52 (123.97) | 235.9 (63.28) |
| LDH (U/L) | NA |  | 1,034 (42.27%) | 1,006 (42.90%) | 28 (27.72%) |
|  | NA |  | 2,446 (100.00%) | 2,345 (100.00%) | 101 (100.00%) |
| Troponin (ng/L) |  | 0.0438 | 109.5 (119.79) | 108.81 (120.98) | 120.57 (98.21) |
|  | NA |  | 1,260 (51.51%) | 1,229 (52.41%) | 31 (30.69%) |
| Creatine kinase (U/L) |  | 0.9721 | 28.77 (67.39) | 29.16 (69.3) | 22.31 (12.58) |
|  | NA |  | 1,935 (79.11%) | 1,863 (79.45%) | 72 (71.29%) |
| Creatine kinase MB (U/L) |  | 0.6977 | 1.47 (3.37) | 1.49 (3.39) | 1.2 (3.01) |
|  | NA |  | 993 (40.60%) | 972 (41.45%) | 21 (20.79%) |
| **Echocardiography** | | | | | |
| Left ventricular end-diastolic diameter (mm) |  | < 0.0001 | 44.21 (6.4) | 44.59 (6.31) | 39.62 (5.64) |
|  | NA |  | 1,958 (80.05%) | 1,894 (80.77%) | 64 (63.37%) |
| Left atrial diameter (mm) |  | 0.0345 | 53.18 (9.42) | 52.87 (9.43) | 58.08 (7.7) |
|  | NA |  | 2,242 (91.66%) | 2,153 (91.81%) | 89 (88.12%) |
| Right ventricular end-diastolic diameter (mm) |  | 0.8965 | 32.41 (5.21) | 32.4 (5.23) | 32.46 (4.92) |
|  | NA |  | 1,956 (79.97%) | 1,892 (80.68%) | 64 (63.37%) |
| Right atrial diameter (mm) |  | < 0.0001 | 53.94 (9.45) | 53.53 (9.45) | 58.92 (7.87) |
|  | NA |  | 1,953 (79.84%) | 1,889 (80.55%) | 64 (63.37%) |
| IVS thickness (mm) |  | < 0.0001 | 15.05 (3.99) | 14.61 (3.68) | 20.46 (3.71) |
|  | NA |  | 1,982 (81.03%) | 1,916 (81.71%) | 66 (65.35%) |
| Tricuspid regurgitation | < moderate | 0.0008 | 572 (23.39%) | 537 (22.87%) | 35 (35.71%) |
|  | ≥ moderate |  | 86 (3.52%) | 71 (3.02%) | 15 (15.31%) |
|  | NA |  | 1,788 (73.1%) | 1,740 (74.11%) | 48 (48.98%) |
| Pulmonary regurgitation | < moderate | 1.0000 | 655 (26.78%) | 605 (25.77%) | 50 (51.02%) |
|  | ≥ moderate |  | 3 (0.12%) | 3 (0.13%) | 0 (0%) |
|  | NA |  | 2,446 (100.00%) | 2,345 (100.00%) | 101 (100.00%) |
| Mitral regurgitation | < moderate | 0.0036 | 571 (23.34%) | 535 (22.79%) | 36 (36.73%) |
|  | ≥ moderate |  | 87 (3.56%) | 73 (3.11%) | 14 (14.29%) |
|  | NA |  | 1,788 (73.1%) | 1,740 (74.11%) | 48 (48.98%) |
| Mitral stenosis | < moderate | 1.0000 | 658 (26.9%) | 608 (25.89%) | 50 (51.02%) |
|  | ≥ moderate |  | 0 (0.00%) | 0 (0.00%) | 0 (0.00%) |
|  | NA |  | 1,788 (73.1%) | 1,740 (74.11%) | 48 (48.98%) |
| Aortic regurgitation | < moderate | 1.0000 | 635 (25.96%) | 587 (25.0%) | 48 (48.98%) |
|  | ≥ moderate |  | 13 (0.53%) | 12 (0.51%) | 1 (1.02%) |
|  | NA |  | 1,798 (73.51%) | 1,749 (74.49%) | 49 (50.0%) |
| Aortic stenosis | < moderate | 0.0638 | 619 (25.31%) | 569 (24.23%) | 50 (51.02%) |
|  | ≥ moderate |  | 39 (1.59%) | 39 (1.66%) | 0 (0%) |
|  | NA |  | 1,788 (73.1%) | 1,740 (74.11%) | 48 (48.98%) |
| Left ventricular function (diastolic) | Normal | < 0.0001 | 30 (1.23%) | 30 (1.28%) | 0 (0%) |
|  | Grade 1 dysfunction |  | 190 (7.77%) | 185 (7.89%) | 5 (4.95%) |
|  | Grade 2 dysfunction |  | 50 (2.04%) | 46 (1.96%) | 4 (3.96%) |
|  | Grade 3 dysfunction |  | 8 (0.33%) | 5 (0.21%) | 3 (2.97%) |
|  | Grade 4 dysfunction |  | 11 (0.45%) | 10 (0.43%) | 1 (0.99%) |
|  | NA |  | 2,157 (88.18%) | 2,069 (88.23%) | 88 (87.13%) |
| Left ventricular function (systolic) | Preserved | < 0.0001 | 352 (14.40%) | 338 (14.42%) | 14 (13.86%) |
|  | Mildly reduced |  | 75 (3.06%) | 62 (2.64%) | 13 (12.87%) |
|  | Moderately reduced |  | 42 (1.71%) | 34 (1.45%) | 8 (8.92%) |
|  | Severely reduced |  | 26 (1.07%) | 24 (1.02%) | 2 (1.98%) |
|  | NA |  | 1,951 (79.76%) | 1,887 (80.47%) | 64 (63.37%) |
| **Cardiac MRI** | | | | | |
| Left ventricular ejection fraction (%) |  | 0.0607 | 54.15 (14.41) | 55.6 (14.89) | 48.79 (10.91) |
|  | NA |  | 2,380 (97.30%) | 2,293 (97.78%) | 87 (86.14%) |
| Right ventricular ejection fraction (%) |  | 0.3764 | 50.85 (12.0) | 51.72 (11.87) | 47.86 (11.97) |
|  | NA |  | 2,384 (97.47%) | 2,297 (97.95%) | 87 (86.14%) |
| Right ventricular end-diastolic diameter (mm) (mm) |  | 0.2882 | 40.71 (6.44) | 41.0 (6.19) | 39.79 (7.11) |
|  | NA |  | 2,387 (97.59%) | 2,300 (98.08%) | 87 (86.14%) |
| Left ventricular end-diastolic volume (mL) |  | 0.5572 | 1,64.75 (52.54) | 164.39 (56.19) | 166.0 (36.97) |
|  | NA |  | 2,383 (97.42%) | 2,296 (97.91%) | 87 (86.14%) |
| Right ventricular end-diastolic volume (mL) |  | 0.3014 | 168.12 (67.56) | 166.21 (71.72) | 174.79 (49.81) |
|  | NA |  | 2,383 (97.42%) | 2,296 (97.91%) | 87 (86.14%) |
| Left ventricular end-systolic volume (mL) |  | 0.1261 | 78.16 (43.31) | 75.92 (46.72) | 86.0 (26.84) |
|  | NA |  | 2,383 (97.42%) | 2,296 (97.91%) | 87 (86.14%) |
| Left ventricular stroke volume (mL) |  | 0.3407 | 86.97 (26.01) | 89.11 (26.74) | 79.93 (22.03) |
|  | NA |  | 2,386 (97.55%) | 2,299 (98.04%) | 87 (86.14%) |
| Right ventricular stroke volume (mL) |  | 0.8888 | 83.75 (27.76) | 84.35 (28.03) | 81.79 (26.74) |
|  | NA |  | 2,386 (97.55%) | 2,299 (98.04%) | 87 (86.14%) |
| Left ventricular cardiac output (L/min) |  | 0.5758 | 5.57 (1.45) | 5.69 (1.47) | 5.19 (1.31) |
|  | NA |  | 2,386 (97.55%) | 2,299 (98.04%) | 87 (86.14%) |
| Right ventricular cardiac output (L/min) |  | 0.8749 | 5.37 (1.53) | 5.36 (1.35) | 5.41 (1.99) |
|  | NA |  | 2,386 (97.55%) | 2,299 (98.04%) | 87 (86.14%) |
| Left ventricular cardiac index (L/min/m²) |  | 0.3467 | 2.94 (0.74) | 3.02 (0.77) | 2.71 (0.58) |
|  | NA |  | 2,395 (97.91%) | 2,308 (98.42%) | 87 (86.14%) |
| Right ventricular cardiac index (L/min/m²) |  | 0.7465 | 2.83 (0.73) | 2.83 (0.65) | 2.83 (0.92) |
|  | NA |  | 2,393 (97.83%) | 2,306 (98.34%) | 87 (86.14%) |
| Left ventricular end-systolic diameter (mm) |  | 0.1053 | 47.57 (6.7) | 48.17 (6.7) | 45.57 (6.3) |
|  | NA |  | 2,386 (97.55%) | 2,299 (98.04%) | 87 (86.14%) |
| Left ventricular end-diastolic diameter (mm) |  | 0.1053 | 47.57 (6.7) | 48.17 (6.7) | 45.57 (6.3) |
|  | NA |  | 2,386 (97.55%) | 2,299 (98.04%) | 87 (86.14%) |
| Extracellular volume (%) |  | 0.5604 | 76.88 (25.59) | 76.68 (26.08) | 78.81 (20.3) |
|  | NA |  | 8,950 (97.60%) | 8,829 (97.80%) | 121 (85.21%) |
| Left ventricular ejection fraction (%) |  | 0.6797 | 5.52 (1.66) | 5.53 (1.71) | 5.51 (1.03) |
|  | NA |  | 8,950 (97.60%) | 8,829 (97.80%) | 121 (85.21%) |
| Right ventricular ejection fraction (%) |  | 0.1508 | 5.05 (1.65) | 5.02 (1.7) | 5.3 (1.14) |
|  | NA |  | 8,950 (97.60%) | 8,829 (97.80%) | 121 (85.21%) |
| Right ventricular end-diastolic diameter (mm) (mm) |  | 0.4452 | 2.95 (0.93) | 2.94 (0.96) | 3.02 (0.52) |
|  | NA |  | 9,008 (98.23%) | 8,880 (98.36%) | 128 (90.14%) |
| Left ventricular end-diastolic volume (mL) |  | 0.1373 | 2.67 (0.87) | 2.65 (0.89) | 2.9 (0.59) |
|  | NA |  | 9,004 (98.19%) | 8,876 (98.32%) | 128 (90.14%) |
| Right ventricular end-diastolic volume (mL) |  | 0.5331 | 47.35 (7.29) | 47.45 (7.11) | 46.45 (8.68) |
|  | NA |  | 8,951 (97.61%) | 8,831 (97.82%) | 120 (84.51%) |
| Left ventricular end-systolic volume (mL) |  | 0.5331 | 47.35 (7.29) | 47.45 (7.11) | 46.45 (8.68) |
|  | NA |  | 8,951 (97.61%) | 8,831 (97.82%) | 120 (84.51%) |
| Left ventricular stroke volume (mL) |  | < 0.0001 | 30.05 (8.61) | 28.83 (6.99) | 43.78 (12.33) |
|  | NA |  | 8,987 (98.00%) | 8,860 (98.14%) | 127 (89.44%) |
