# Supplementary material for: Screening for patients at risk for cardiac amyloidosis via electronic health records: A multicenter machine learning development and validation study
Source: PLOS Digit Health. 2026 Oct 8;5(10):e0001637. doi: 10.1371/journal.pdig.0001637 (PMC13649026; doi:10.1371/journal.pdig.0001637)
Supplement: Supplementary file 3 [file pdig.0001637.s003.docx]

| Parameter | Parameter value | *p* value | Entire data | No high-grade uptake | High-grade uptake |
| --- | --- | --- | --- | --- | --- |
| Number of patients, n (%) |  |  | 1521 (100.00%) | 1376 (90.47%) | 145 (9.53%) |
| High-grade cardiac uptake (Perugini grade 2 or 3) |  | < 0.0001 | 145 (9.53%) | 0 (0%) | 145 (100.0%) |
| Male sex |  | < 0.0001 | 639 (42.01%) | 517 (37.57%) | 122 (84.14%) |
|  | NA |  | 9 (0.59%) | 7 (0.51%) | 2 (1.38%) |
| Carpal tunnel syndrome |  | < 0.0001 | 110 (7.23%) | 45 (3.27%) | 65 (44.83%) |
| Atrial fibrillation |  | < 0.0001 | 374 (24.59%) | 270 (19.62%) | 104 (71.72%) |
|  | NA |  | 23 (1.51%) | 21 (1.53%) | 2 (1.38%) |
| Interventricular septum thickness (mm) |  | < 0.0001 | 13.52 (4.53) | 12.79 (4.1) | 17.64 (4.62) |
|  | NA |  | 1143 (75.15%) | 1055 (76.67%) | 88 (60.69%) |
| Left ventricular function (systolic) | Preserved | 0.00984 | 190 (12.49%) | 172 (12.5%) | 18 (12.41%) |
|  | Mildly reduced |  | 67 (4.4%) | 54 (3.92%) | 13 (8.97%) |
|  | Moderately reduced |  | 74 (4.87%) | 57 (4.14%) | 17 (11.72%) |
|  | Severely reduced |  | 10 (0.66%) | 10 (0.73%) | 0 (0%) |
|  | NA |  | 1180 (77.58%) | 1083 (78.71%) | 97 (66.9%) |
