# Supplementary material for: Screening for patients at risk for cardiac amyloidosis via electronic health records: A multicenter machine learning development and validation study
Source: PLOS Digit Health. 2026 Oct 8;5(10):e0001637. doi: 10.1371/journal.pdig.0001637 (PMC13649026; doi:10.1371/journal.pdig.0001637)
Supplement: Supplementary file 4 [file pdig.0001637.s004.docx]

| Model | Cohort | Subgroup | AUC | SNS | SPC | PPV | NPV |
| --- | --- | --- | --- | --- | --- | --- | --- |
| Amylo-Detect | Cross-validation | All | **0.93**  **(0.91; 0.95)** | **0.71 (0.63; 0.79)** | **0.93 (0.92; 0.93)** | **0.14 (0.12; 0.15)** | **1.00 (0.99; 1.00)** |
| Davies et al. score 2022 | Cross-validation | All | 0.55  (0.51; 0.59) | 0.50  (0.42; 0.58) | 0.60  (0.59; 0.61) | 0.02  (0.02; 0.02) | 0.99  (0.99; 0.99) |
| Amylo-Detect | Independent validation | All | **0.91 (0.88; 0.94)** | 0.64 (0.55; 0.74) | **0.94 (0.93; 0.95)** | **0.32 (0.28; 0.37)** | **0.98 (0.98; 0.99)** |
| Davies et al. score 2022 | Independent validation | All | 0.59  (0.54; 0.64) | 0.64  (0.55; 0.73) | 0.54 (0.52; 0.56) | 0.06 (0.05; 0.07) | 0.97 (0.97; 0.98) |
| Amylo-Detect | Cross-validation | ECV >40% (n=16) | **0.84**  **(0.64; 1.00)** | **1.00**  **(1.00; 1.00)** | 0.43  (0.14; 0.86) | **0.69**  **(0.60; 0.90)** | **1.00**  **(1.00; 1.00)** |
| Davies et al. score 2022 | Cross-validation | ECV >40% (n=16) | 0.72  (0.62; 0.82) | 0.48  (0.29; 0.67) | **0.97**  **(0.95; 0.98)** | 0.40  (0.24; 0.57) | 0.98  (0.97; 0.99) |
| Amylo-Detect | Independent validation | ECV >40% (n=14) | **1.00**  **(1.00; 1.00)** | **1.00**  **(1.00; 1.00)** | NA | **0.79**  **(0.79; 0.79)** | NA |
| Davies et al. score 2022 | Independent validation | ECV >40% (n=14) | 0.83  (0.69; 0.95) | 0.75  (0.50; 1.00) | 0.91  (0.82; 0.98) | 0.69  (0.50; 0.92) | 0.93  (0.87; 1.00) |
