# Supplementary material for: Screening for patients at risk for cardiac amyloidosis via electronic health records: A multicenter machine learning development and validation study
Source: PLOS Digit Health. 2026 Oct 8;5(10):e0001637. doi: 10.1371/journal.pdig.0001637 (PMC13649026; doi:10.1371/journal.pdig.0001637)
Supplement: Supplementary file 15 [file pdig.0001637.s016.docx]

**Response to Reviewers**

**Screening for patients at risk for cardiac amyloidosis via electronic health records: A multicenter machine learning development and validation study**

Manuscript ID PDIG-D-26-00090. Submitted to PLOS Digital Health.

We thank the editor and both reviewers for the constructive and detailed assessment. The comments have materially improved the manuscript. Reviewer comments are shown in **bold**. New or modified manuscript text is reproduced here in *blue italics*. Tracked changes appear in the revised manuscript and supplement.

# **Reviewer 1**

**Summary**

**In this study, the authors use a trained machine learning algorithm to detect patients with cardiac amyloidosis from electronic health records. Treatment of cardiac amyloidosis has developed rapidly in recent years with nuclear imaging and new medical interventions. Still, the main issue is early disease detection to enable timely referral of patients to diagnostic imaging and therapy. Therefore, this is clinically meaningful and important field of research. I have some further comments below.**

**Comments 1.1: Authors have developed an early detection algorithm for cardiac ATTR amyloidosis (Perugini grade 2 or 3). However, the early ATTR might present as Perugini grade 1 uptake on scintigraphy. This will impact the algorithm's training, since the earliest disease may be missed. Positive algorithm findings might be classified as false positives due to less than grade 2 uptake.**

**Response:** We thank the reviewer for raising this important question. We agree that Perugini grade 1 uptake can occasionally reflect early ATTR, although it is non-specific and not by itself diagnostic, which is why current diagnostic pathways reserve a non-biopsy ATTR diagnosis for grade 2-3 uptake with negative monoclonal screening^1^. Our primary endpoint (grade ≥2) therefore reflects the clinically actionable referral target the tool is designed to support.

To address the potential influence of grade 1 uptake, we performed a sensitivity analysis in all three cohorts. For a prediction target of Perugini grade ≥1, the model retained good discrimination (AUC 0.85 in the development cross-validation cohort, 0.89 in the independent internal validation cohort), and 0.93 in the external validation cohort compared with AUC 0.93, 0.91 and 0.91 for the primary grade ≥2 endpoint for the same cohorts, respectively. While the sensitivity, particularly for the development cohort, was considerably lower than in the primary analysis, the overall results are excellent considering that the model was trained to detect patients with grade ≥2 rather than grade ≥1.

We added these findings to the Results section and presented them in Supplemental Figure S7.

*Lastly, a sensitivity analysis was performed to evaluate the detection of patients with any cardiac uptake (Perugini grade ≥1), using the same model, trained to detect Perugini grade ≥2 cardiac uptake. The model achieved an AUC of 0.86 (95% CI 0.85; 0.88) in the development AUC 0.90 (95% CI 0.87; 0.92) in the independent internal validation cohort and AUC 0.94 (95% CI 0.91; 0.96) in the external validation cohort (Supplemental Figure S7).*

***Figure S7.*** *Diagnostic performance for any cardiac uptake (Perugini grade ≥1).*

**Comment 1.2: Patients with cardiac amyloidosis frequently have multiple health care contacts prior to the correct diagnosis, and the diagnostic delay is long. This is an opportunity for the algorithm to detect the disease (there should be lots of data in positive cases). I would emphasise that there is frequently a lot of EHR data available prior to the correct diagnosis (for example, study: https://doi.org/10.1161/CIRCULATIONAHA.118.038169)**

**Response:** We agree and now make this point in the Discussion, citing the suggested work. The long pre-diagnostic interval is precisely the window Amylo-Detect targets.

*CA is typically preceded by a prolonged diagnostic interval with multiple healthcare encounters, during which extracardiac features accumulate in the EHRs21. These records are exploited by Amylo-Detect to raise suspicion among patients with features consistent with a scintigraphy-referral population.*

**Comment 1.3: The median age of the development cohort was (64 y, 52-73 y). This is quite a low age range for ATTR cardiomyopathy patients. Does this affect the algorithm's training? Since older patients' electronic health record data might differ from that of younger patients? How common is the hereditary form in Austria among younger patients?**

**Response:** The all-comer scintigraphy cohort is younger than a pure ATTR-CM population because bone scintigraphy is commonly performed for non-amyloid indications. Patients with high-grade uptake were older (median age 79 in development cohort), consistent with predominantly wild-type ATTR. Hereditary ATTR is uncommon in Austria, so the older skew of the positive cases largely reflects wild-type disease. The breadth of age supports generalizability rather than harming training. We have added a clarifying statement to the Population Characteristics section within the Results. The ability of Amylo-Detect to identify cardiac amyloidosis is demonstrated in the subgroup analysis (Figure 2), where performance was robust for patients younger than 70 years (AUC 0.97) or patients at least 70 years old (AUC 0.92).

*Patients with high-grade uptake were considerably older than the overall cohort (median age 79 [IQR 71-87] years in development), consistent with predominantly wild-type ATTR-CM in this population.*

**Comment 1.4: The authors developed a model with a high AUC. Another approach would be to develop a model with very high sensitivity to detect all patients with an elevated risk of ATTR. Further screening with SPECT is quite affordable. This would increase the yield of the ATTR detection but would demand more resources for follow-up nuclear imaging. Is this approach be possible from a practical / economic point of view?**

**Response:** Thank you for this question. This is a useful framing and aligns with our intended use as a prioritization tool. Because Amylo-Detect outputs a continuous probability (rather than solely a yes/no-prediction), the threshold can be lowered to prioritize sensitivity.

We now report a sensitivity-prioritized operating point alongside the balanced operating point, using a single fixed threshold selected on the development cohort and applied unchanged to both validation cohorts.

At the sensitivity-prioritized threshold, Amylo-Detect achieved a sensitivity of 0.90, 0.86 and 0.94 in the cross-validation, independent internal and external cohorts, respectively, with negative predictive value ≥0.99 in all cohorts. As expected at a screening threshold in a low-prevalence setting (high-grade uptake 3.0%), positive predictive value was modest (0.08 to 0.25), corresponding to roughly 4 to 12 scans per additional case identified. Given that bone scintigraphy is inexpensive, widely available and carries low radiation exposure as well as considering that disease-modifying ATTR therapy is most effective when initiated early, this trade-off may be favorable for centers prioritizing high detection rates over cost-effectiveness. The consistently high negative predictive value supports the model's value as a rule-out and prioritization aid. We have added the results related to these operating points and their practical implications to the Results and Discussion.

Added statement in Results:
*At a sensitivity-prioritized threshold (0.014), defined by lowering the cut-off until sensitivity reached 0.90 in the development cohort, Amylo-Detect maintained high sensitivity across all cohorts (development 0.90, 95% CI 0.85 to 0.94; internal validation 0.86, 95% CI 0.79 to 0.93; external validation 0.94, 95% CI 0.90 to 0.97). Negative predictive value remained close to optimal (development 1.00, 95% CI 1.00 to 1.00; internal validation 0.99, 95% CI 0.99 to 1.00; external validation 0.99, 95% CI 0.99 to 1.00). As expected, given the low prevalence of high-grade uptake (3.0%), positive predictive value at a screening threshold was modest (development 0.08, 95% CI 0.07 to 0.08; internal validation 0.14, 95% CI 0.13 to 0.16; external validation 0.25, 95% CI 0.24 to 0.27), corresponding to approximately 4 to 13 confirmatory scans per additional case identified. Specificity at this operating point was 0.83 (95% CI 0.82 to 0.83), 0.78 (95% CI 0.76 to 0.79) and 0.71 (95% CI 0.68 to 0.73), respectively.*

Added statement in Discussion:
*The optimal operating point depends on local resource constraints and the relative cost of a missed diagnosis. A sensitivity-prioritized threshold increases downstream confirmatory imaging but maximizes detection of treatable disease, whereas a balanced threshold reduces scan volume at the cost of some missed cases. Centers can position this threshold according to clinical, operational and economic preferences.*

**Comment 1.5: The algorithm relies on the electronic health record data. In health care systems with poor communication between hospitals or problems integrating primary healthcare and cardiology clinic data, this kind of algorithms value might be limited. Could the authors comment on how this approach would work, for example, in the United States, in a healthcare setting with multiple private healthcare providers?**

**Response:** We agree this is a potential constraint and have added it as a limitation. Amylo-Detect requires structured data to be accessible at the point of inference. Performance in fragmented or poorly interoperable systems is unknown and would need local validation. We generally intend the system to be used at hospitals or larger centers where electronic health records are integrated across departments, making the availability of the models’ relatively commonly assessed parameters likely. We also note the model is robust to extensive missingness, which partly mitigates incomplete records.

*Amylo-Detect requires structured electronic health record data to be retrievable at the point of inference. In fragmented systems with limited interoperability between primary care, hospitals and cardiology services, the available data may be sparser than in our integrated setting, and local validation would be required before deployment.*

**Comment 1.6: Another limitation is the use of echocardiography data in the algorithm. Would the patient need a prior cardiology clinic visit to be detected by this algorithm?**

**Response:** Thank you for this question. Echocardiographic assessment can contribute to the model but is not required. The model is robust when echocardiographic variables are entirely missing (AUC 0.96; Figure 2). The two single most important predictors (carpal tunnel syndrome and atrial fibrillation) do not require echocardiography (and the model is also robust if either of them is missing as shown in Figure 2).

*We show that this approach yields excellent performance across three large-scale cohorts and several clinically relevant subgroups, demonstrating that Amylo-Detect is robust to missingness of individual parameters, including echocardiographic findings.*

**Comment 1.7: Could authors characterize the features of patients detected by Amylo-Detect and missed by the clinical standard? Was there some clinician bias to age, gender or was it more frequently early disease harder to detect?**

**Response:** We thank the reviewer for this question. We have performed a comparison of clinical characteristics of patients who were missed by clinical assessment versus those who were correctly detected. The analysis was performed in the two Vienna cohorts, where comprehensive parameters, beyond those required by Amylo-Detect, were present. Patients not detected as part of clinical routine had lower bilirubin values (p=0.046), were less commonly affected by carpal tunnel syndrome (p=0.03) and had significantly lower hemoglobin (p=0.02) and hematocrit (p=0.049) values. Otherwise, clinical characteristics did not show a significant difference. We added a corresponding statement to the Results.
*Among patients with CA-suggestive uptake missed in clinical routine referral, patients had markedly lower bilirubin values (p=0.046), were less commonly affected by carpal tunnel syndrome (p=0.03) and had significantly lower hemoglobin (p=0.02) and hematocrit (p=0.049) values in comparison with patients correctly identified by routine clinical suspicion. Other clinical characteristics were comparable between groups.*

# **Reviewer 2**

**Summary**

**This manuscript presents Amylo-Detect, an ML model designed to identify patients at risk of CA using routinely available structured EHR data. The model was developed in a large retrospective cohort of consecutive patients referred for DPD bone scintigraphy at Vienna General Hospital and validated in both a temporally separated internal cohort and an external cohort from University Hospital Essen. The model predicts high-grade cardiac uptake on scintigraphy, defined as Perugini grade ≥2, as a marker suggestive of CA.**

**The study includes 9,170 patients in the development cohort, 2,446 in the internal validation cohort, and 1,521 in the external validation cohort. High-grade cardiac uptake was present in 388 patients overall. Amylo-Detect achieved strong discrimination, with AUCs of 0.93 in development, 0.91 in internal validation, and 0.91 in external validation. The authors report that the model outperformed an existing Mayo ATTR-CM score and clinical referral indication in several metrics. They also report that model-positive status was associated with all-cause mortality and heart failure hospitalisation.**

**The manuscript addresses an important clinical problem. CA remains underdiagnosed, and timely identification of patients suitable for confirmatory testing is a major clinical bottleneck. The study has several strengths, including the large sample size, temporal internal validation, external validation, use of structured EHR data, retention of patients with missing data, comparison with existing approaches, and public availability of a web application.**

**However, several issues need to be addressed before the conclusions are fully supported. Most importantly, the model was developed and validated only in patients already referred for bone scintigraphy, yet parts of the manuscript imply applicability to broader hospital-wide EHR screening. The primary outcome is CA-suggestive uptake rather than confirmed CA. The clinical utility of the selected threshold is not yet adequately demonstrated, especially given lower sensitivity than clinical referral indication. Further clarity is also needed around missing data handling, calibration, threshold selection, external validation using limited variables, and safe implementation of the public web app.**

**Major comments**

**Comment 2.1: The intended-use population is broader than the evidence supports**

**The manuscript frames Amylo-Detect as a tool for identifying patients at risk of CA using EHR data. However, all development and validation cohorts consisted of patients who had already been referred for DPD bone scintigraphy. This is a highly selected population. Even if the cohort includes "all-comer referrals" for scintigraphy, it does not represent an unselected hospital, cardiology, heart failure, aortic stenosis, primary care, or general EHR population.**

**This distinction is central to the clinical interpretation of the work. A model that performs well among patients already selected for scintigraphy may not perform similarly if deployed across all hospital patients. The statement that the results apply to "all patients visiting a hospital" is therefore not supported by the current study design.**

**Suggested revision: The authors should define the intended-use population more narrowly throughout the manuscript. For example, the model could be framed as identifying patients at risk of CA-suggestive scintigraphy findings among patients with clinical features similar to those entering a scintigraphy-referral population. Alternatively, broader claims would require validation in an unselected EHR population with systematic outcome ascertainment.**

**Response:** We agree and have narrowed the framing throughout. The model is now defined as identifying patients at risk of cardiac-amyloidosis-suggestive uptake among patients with clinical features consistent with a scintigraphy-referral population. We deleted the statement that the results apply to all patients visiting a hospital and removed the claim that the diversity of the cohort supports screening of a general hospital population. We now state explicitly that the model was developed and validated in a scintigraphy-referral cohort and that broader hospital-wide screening would require prospective validation in an unselected population with systematic ascertainment of cardiac amyloidosis.

We adapted the manuscript throughout and removed statements such as: *’This analysis indicates that our results apply to the proposed target population (all patients visiting a hospital)’* and *’This diversity supports the potential generalizability of the model to real-world EHR populations.’*

We further replaced the previous statement in the discussion, stating that the diverse study population characteristics support the ability of Amylo-Detect to screen through the general hospital population and now explicitly highlight that the model was trained and validated in a cohort of scintigraphy referrals.

*We show that Amylo-Detect can identify patients at risk of CA-suggestive scintigraphy uptake among patients whose clinical features resemble those of a bone scintigraphy referral population. Although the study population spanned a wide range of clinical indications beyond suspected CA and a wide range of ages and comorbidities, this study was neither developed nor validated in an unselected hospital and broader hospital-wide screening requires validation in a prospective study with systematic assessment of cardiac amyloidosis in all patients.*

**Comment 2.2: Clinical utility is not yet adequately demonstrated**

**The model demonstrates high AUC, but AUC alone is insufficient to establish clinical usefulness for a screening or referral tool. At the selected operating threshold, Amylo-Detect has lower sensitivity than clinical standard in both validation cohorts. In the internal validation cohort, sensitivity is 0.64 for Amylo-Detect vs. 0.88 for clinical standard. In the external validation cohort, sensitivity is 0.73 vs. 0.97.**

**This is particularly important because the proposed use case is screening or referral support for a progressive and treatable disease. Missing cases may be clinically consequential. The model has higher specificity and PPV than clinical standard, but the trade-off needs to be explicitly justified.**

**The PPV is also modest, especially in development, where PPV is 0.14. This may be acceptable if the downstream pathway is low burden and cost-effective, but the manuscript does not quantify the number of additional referrals, scans, monoclonal protein tests, false positives, or downstream resource implications.**

**Suggested revision: The authors should provide decision-curve analysis, net benefit, number needed to screen, number needed to scan, false-positive burden, and threshold-specific performance across clinically plausible thresholds. They should explicitly state whether Amylo-Detect is intended to replace clinical judgement, supplement clinical judgement, or prioritise patients for expert review.**

**Response:**

We thank the reviewer for this important comment and agree that discrimination alone does not establish clinical usefulness. We have added the requested analyses and clarified the intended use. Amylo-Detect supplements and prioritizes and does not replace clinical routine suspicion-based referral for scintigraphy. The current threshold favors efficient rule-in, is adjustable, and the added decision-curve, threshold-specific, number-needed-to-scan, and false-positive-burden analyses demonstrate net clinical benefit over clinical routine suspicion in this population across the relevant threshold range while substantially reducing unnecessary downstream testing.

We have added an explicit statement regarding intended use to the Discussion:

*When employed to improve referral for bone scintigraphy, Amylo-Detect is intended to supplement clinical judgment and to prioritize patients for confirmatory bone scintigraphy and monoclonal protein testing. It is not intended to replace clinical assessment. In deployment, it operates alongside routine clinical suspicion and does not override it, so a patient who is clinically suspected of cardiac amyloidosis is referred regardless of the model output. The model therefore adds rule-in capacity for patients not already flagged by routine and does not remove patients from the existing pathway.*

The sensitivity trade-off is intentional and we added an explicit justification to the manuscript:

*At an operating point of 0.043, sensitivity is lower than clinical routine (0.71 vs 0.84 development, 0.64 vs 0.88 internal validation, 0.73 vs 0.97 external validation), while specificity and PPV are consistently higher (specificity 0.93 vs 0.88, 0.94 vs 0.79, 0.88 vs 0.73; PPV 0.14 vs 0.13, 0.32 vs 0.15, 0.39 vs 0.27). The threshold is set to favor rule-in efficiency. When applied in a clinical routine scenario, as the model supplements rather than replaces clinical suspicion, the operationally relevant sensitivity is that of model plus routine combined, which exceeds either alone.*

We have added a clarifying statement that 0.043 (recalibrated from 0.77) is a single threshold applied across all cohorts for the primary results and is explicitly adaptable to the clinical scenario and clinician preference. We further specify that the web app returns the continuous probability-like score rather than a fixed label.

*Importantly, by outputting a continuous probability score, Amylo-Detect allows for the selection of center-specific and clinical context-specific cut-offs. […] The web app supports this concept by providing the output as a probability-like score rather than a fixed label.*

To support site- and scenario-specific operating points, we now provide threshold-specific sensitivity, specificity, PPV and NPV across the full plausible range (**Supplemental Figure S8**), allowing users to select an operating point matched to their tolerance for missed cases.

***Supplemental Figure S8****. Performance metrics over various thresholds.*

We further added an the performance metrics of Amylo-Detect at a sensitivity-prioritized threshold and added the corresponding findings to the Results as specifically requested by Reviewer 1:
*At a sensitivity-prioritized threshold (0.014), defined by lowering the cut-off until sensitivity reached 0.90 in the development cohort, Amylo-Detect maintained high sensitivity across all cohorts (development 0.90, 95% CI 0.85 to 0.94; internal validation 0.86, 95% CI 0.79 to 0.93; external validation 0.94, 95% CI 0.90 to 0.97). Negative predictive value remained close to optimal (development 1.00, 95% CI 1.00 to 1.00; internal validation 0.99, 95% CI 0.99 to 1.00; external validation 0.99, 95% CI 0.99 to 1.00). As expected, given the low prevalence of high-grade uptake (3.0%), positive predictive value at a screening threshold was modest (development 0.08, 95% CI 0.07 to 0.08; internal validation 0.14, 95% CI 0.13 to 0.16; external validation 0.25, 95% CI 0.24 to 0.27), corresponding to approximately 4 to 13 confirmatory scans per additional case identified. Specificity at this operating point was 0.83 (95% CI 0.82 to 0.83), 0.78 (95% CI 0.76 to 0.79) and 0.71 (95% CI 0.68 to 0.73), respectively.*

Across clinically plausible threshold probabilities, the net benefit of Amylo-Detect at the operating point exceeded that of clinical routine from pt ~0.06 (development) and pt ~0.07 (internal validation). In external validation, routine suspicion is already highly sensitive (0.97) but poorly specific (0.73), and net benefit exceeds routine from pt ~0.14 upward. We added a new section in the Results, ‘Clinical Utility’, where we summarize findings related to the decision curve analysis and downstream burden. We further added a new Figure, **Supplemental Figure S9** and a corresponding reference in the Clinical Utility section.

*To assess clinical usefulness beyond discrimination, we performed decision-curve analysis and quantified the downstream testing burden at the operating point (threshold 0.043).*

*Decision-curve analysis (Supplemental Figure S9) showed that the net benefit of Amylo-Detect at the operating point exceeded that of clinical routine from a threshold probability of approximately 0.06 in development and 0.07 in internal validation. In external validation, where routine suspicion was highly sensitive (0.97) but poorly specific (0.73), net benefit of Amylo-Detect exceeded that of clinical routine from a threshold probability of approximately 0.14 upward.*

***Supplemental Figure S9****. Decision curve analysis.*

We have added an extensive quantification of downstream burden to the new Clinical Utility section.

*At the operating point, the number needed to scan to identify one patient with high-grade uptake was 7.1, 3.1, and 2.6 in development, internal validation, and external validation, compared with 7.7, 6.7, and 3.7 for clinical routine. False positives per 1000 patients screened were 69, 58, and 109 for Amylo-Detect versus 118, 201, and 244 for clinical routine. The number needed to screen to identify one case was 88, 38, and 14 across the three cohorts. In external validation, application of Amylo-Detect corresponded to approximately 178 patients per 1000 referred for confirmatory scintigraphy and monoclonal protein testing, compared with 336 per 1000 under clinical routine, at a sensitivity of 0.73 versus 0.97.*

**Comment 2.3: The comparison with "clinical standard" needs clearer framing**

**The comparison with clinical routine is interesting but potentially misleading. "Clinical standard" appears to be inferred from the referral indication among patients who already underwent scintigraphy. This is not the same as evaluating clinician decision-making in an unselected EHR population. Rather, it assesses whether the indication for an already-performed scan included suspected CA.**

**There is also a conceptual issue with reporting AUC for a binary clinical referral indication and comparing this with a continuous model output. The authors should explain exactly how this AUC was calculated and whether this is a fair comparison.**

**Suggested revision: The authors should reframe this comparator as "referral indication among scintigraphy recipients" rather than "clinical standard" unless they can provide a more direct comparator to real-world clinician referral decisions.**

**Response:** We have reframed the comparator as clinical routine suspicion for cardiac amyloidosis and explicitly mention that this in among patients referred for scintigraphy due to any indication. We also clarify the metric. The referral indication is binary, so its receiver-operating-characteristic representation reduces to a single operating point and the corresponding area equals the mean of sensitivity and specificity. The fair head-to-head comparison is therefore at the level of sensitivity, specificity and predictive values, which we report. We retain the AUC value only as a summary and label it accordingly.

*AUC for clinical suspicion was calculated at a single operating point, as the arithmetic mean of sensitivity and specificity. This analysis was performed among patients who were assessed for CA-suggestive uptake via scintigraphy.*

And we added an explicit statement about the non-comparability between the AUCs of Amylo-Detect and routine clinical judgement: *A direct comparison for AUC cannot be made since clinical suspicion only allows measurement at a single operating point.*

**Comment 2.4: Missing data handling is central and requires more transparency**

**The manuscript states that patients had a median of 38 missing parameters out of 50, and 6 missing parameters among the 13 selected model parameters. This level of missingness is substantial. The authors appropriately attempt to handle this using imputation, but more detail is required.**

**The finding that the model performed slightly better in patients with more missing values is potentially reassuring, but it may also suggest that missingness itself is informative. Missingness may encode clinical workflow, investigation availability, institutional practice, or referral patterns rather than disease biology.**

**External validation was apparently performed using only the six most important parameters. This raises further questions about the true input requirements of the deployed model. It is unclear whether the model requires 50 variables, 13 variables, six variables, or can operate meaningfully with almost no variables.**

**Suggested revision: Please provide a detailed missingness table by variable and cohort, clarify whether missingness indicators were used, and describe the KNN imputation process in greater detail. The authors should report performance in patients with complete data, patients with only the six key variables, patients without echocardiographic data, and patients without carpal tunnel syndrome coding. They should also discuss whether missingness patterns may encode local clinical workflows.**

**Response:** Thank you for this question. We expanded the imputation description. Imputation used the k-nearest-neighbours algorithm fitted on the training data only, with 10 neighbors, Euclidean distance and distance weighting via their inverse distance.

We did not add explicit missingness indicators, as this would cause the model to potentially predict based on missingness patterns. On the finding of slightly higher performance with more missing values, we now explain it rather than leaving it implicit. The most informative predictors (carpal tunnel syndrome, atrial fibrillation, sex and age) are rarely missing, whereas extensively investigated patients form a clinically heterogeneous group. We present this as a robustness observation and not as evidence that missingness is desirable.

We report performance among subgroups of patients with more than 6 missing values (AUC 0.94) and with fewer (AUC 0.91), we report performance among patients without echocardiographic data (AUC 0.96) and among patients without carpal tunnel syndrome (AUC 0.96). All subgroup analysis results are shown either in the ‘Subgroup and robustness analyses’ section of the Results or in Table 2.

We report detailed missingness for all three cohorts, for the entire cohort, patients with and without cardiac amyloidosis-suggestive uptake for all parameters in the corresponding clinical characteristics tables, Supplemental Tables S1, S2 and S3.

*Missing values were imputed using k-nearest-neighbours imputation (k=10, Euclidean distance, distance weighting using inverse distance) fitted exclusively on the training data and then applied unchanged to the validation cohorts. No explicit missingness-indicator variables were added as missingness in routine records may encode local workflow, investigation availability and referral practice rather than disease biology. Predictions in patients with extensive missingness should be interpreted with corresponding caution.*

**Comment 2.5: Calibration and threshold selection require stronger reporting**

**Amylo-Detect is presented as providing a continuous probability score, yet calibration reporting is limited. The manuscript states that risk calibration yielded a Brier score of 0.085 and did not improve with isotonic regression. In a low-prevalence screening setting, this is insufficient to establish that the predicted probabilities are clinically interpretable.**

**The probability threshold of 0.77 is described as achieving a "reasonable balance" of diagnostic metrics, but this threshold does not appear to be clinically justified. For a screening/referral tool, threshold choice should be linked to intended use, acceptable false-positive burden, acceptable false-negative rate, and downstream testing capacity.**

**Suggested revision: Please provide calibration plots for all cohorts, calibration slope and intercept, observed versus predicted risk by decile, precision-recall curves, and threshold-specific diagnostic performance tables. Precision-recall analysis is particularly relevant given the low prevalence of the outcome.**

**Response:** We thank the reviewer very much for this comment. During reassessment, we identified an error causing miscalibration (calibration performed after SMOTE balancing) and the corresponding Supplemental Figure to be incorrect. Consequentially, we corrected the existing Figure and replaced it with a corrected version including all cohorts. Calibration continuous to only be fitted on the development cohort and applied unchanged to the other cohorts. The model is now well calibrated, with slope close to 1 and intercept close to 0 and a Brier score of 0.012 in the development cohort. We adapted the employed thresholds accordingly (0.77 to 0.043 and 0.38 to 0.014). Curves were obtained by locally weighted scatterplot smoothing of the binary outcome on the predicted probability.

***Figure S4****. Calibration of Amylo-Detect for all three cohorts before and after logistic recalibration using the development cohort. Recalibration metrics are provided for the recalibrated model.*

As requested, we additionally computed performance across a range of thresholds. Given the already comprehensive content of the manuscript, we feel that including these results would risk overloading the reader with information. Should the reviewer prefer that these tables be included, we would of course be happy to add them.

| **threshold** | **cohort** | **cohort prevalence** | **sensitivity** | **specificity** | **PPV** | **NPV** |
| --- | --- | --- | --- | --- | --- | --- |
| 0.05 | development | 0.016 | 0.662 | 0.947 | 0.167 | 0.994 |
| 0.1 | development | 0.016 | 0.483 | 0.978 | 0.260 | 0.992 |
| 0.15 | development | 0.016 | 0.414 | 0.986 | 0.330 | 0.991 |
| 0.2 | development | 0.016 | 0.359 | 0.992 | 0.413 | 0.990 |
| 0.3 | development | 0.016 | 0.303 | 0.995 | 0.506 | 0.989 |
| 0.5 | development | 0.016 | 0.186 | 0.999 | 0.692 | 0.987 |
| 0.04 | development | 0.016 | 0.676 | 0.944 | 0.163 | 0.995 |
| 0.05 | internal validation | 0.041 | 0.624 | 0.951 | 0.354 | 0.983 |
| 0.1 | internal validation | 0.041 | 0.495 | 0.980 | 0.510 | 0.978 |
| 0.15 | internal validation | 0.041 | 0.455 | 0.989 | 0.648 | 0.977 |
| 0.2 | internal validation | 0.041 | 0.376 | 0.994 | 0.731 | 0.974 |
| 0.3 | internal validation | 0.041 | 0.327 | 0.997 | 0.805 | 0.972 |
| 0.5 | internal validation | 0.041 | 0.257 | 0.999 | 0.897 | 0.969 |
| 0.0432 | internal validation | 0.041 | 0.644 | 0.941 | 0.320 | 0.984 |
| 0.05 | external validation | 0.095 | 0.697 | 0.919 | 0.476 | 0.966 |
| 0.1 | external validation | 0.095 | 0.497 | 0.964 | 0.590 | 0.948 |
| 0.15 | external validation | 0.095 | 0.448 | 0.977 | 0.677 | 0.944 |
| 0.2 | external validation | 0.095 | 0.407 | 0.981 | 0.694 | 0.940 |
| 0.3 | external validation | 0.095 | 0.352 | 0.983 | 0.680 | 0.935 |
| 0.5 | external validation | 0.095 | 0.269 | 0.987 | 0.684 | 0.928 |
| 0.0429 | external validation | 0.095 | 0.731 | 0.906 | 0.451 | 0.970 |

| **threshold** | **cohort** | **NNS per case detected** | **NNScan per case confirmed** | **FP per TP** | **FP per 1000 screened** | **scans per 1000 screened** |
| --- | --- | --- | --- | --- | --- | --- |
| 0.05 | development | 95.521 | 6.000 | 5.000 | 52.345 | 62.814 |
| 0.1 | development | 131.000 | 3.843 | 2.843 | 21.701 | 29.335 |
| 0.15 | development | 152.833 | 3.033 | 2.033 | 13.304 | 19.847 |
| 0.2 | development | 176.346 | 2.423 | 1.423 | 8.070 | 13.740 |
| 0.3 | development | 208.409 | 1.977 | 0.977 | 4.689 | 9.487 |
| 0.5 | development | 339.630 | 1.444 | 0.444 | 1.309 | 4.253 |
| 0.04 | development | 93.571 | 6.122 | 5.122 | 54.744 | 65.431 |
| 0.05 | internal validation | 38.825 | 2.825 | 1.825 | 47.016 | 72.772 |
| 0.1 | internal validation | 48.920 | 1.960 | 0.960 | 19.624 | 40.065 |
| 0.15 | internal validation | 53.174 | 1.543 | 0.543 | 10.221 | 29.027 |
| 0.2 | internal validation | 64.368 | 1.368 | 0.368 | 5.724 | 21.259 |
| 0.3 | internal validation | 74.121 | 1.242 | 0.242 | 3.271 | 16.762 |
| 0.5 | internal validation | 94.077 | 1.115 | 0.115 | 1.226 | 11.856 |
| 0.0432 | internal validation | 37.631 | 3.123 | 2.123 | 56.419 | 82.993 |
| 0.05 | external validation | 15.059 | 2.099 | 1.099 | 72.978 | 139.382 |
| 0.1 | external validation | 21.125 | 1.694 | 0.694 | 32.873 | 80.210 |
| 0.15 | external validation | 23.400 | 1.477 | 0.477 | 20.381 | 63.116 |
| 0.2 | external validation | 25.780 | 1.441 | 0.441 | 17.094 | 55.884 |
| 0.3 | external validation | 29.824 | 1.471 | 0.471 | 15.779 | 49.310 |
| 0.5 | external validation | 39.000 | 1.462 | 0.462 | 11.834 | 37.475 |
| 0.0429 | external validation | 14.349 | 2.217 | 1.217 | 84.813 | 154.504 |

We further provide precision-recall tradeoffs for all cohorts now as AUPRC in the main performance table of the manuscript:

| *Approach* | *Cohort* | *AUC*  *(95% CI)* | *ACC (95% CI)* | *AUPRC (95% CI)* | *SNS*  *(95% CI)* | *SPC*  *(95% CI)* | *PPV*  *(95% CI)* | *NPV*  *(95% CI)* |
| --- | --- | --- | --- | --- | --- | --- | --- | --- |
| *Amylo-Detect* | *Development (cross-validation)* | ***0.93***  ***(0.91; 0.95)*** | ***0.93 (0.92; 0.93)*** | ***0.31 (0.24; 0.39)*** | *0.71 (0.63; 0.79)* | ***0.93 (0.92; 0.93)*** | ***0.14 (0.12; 0.15)*** | *1.00 (0.99; 1.00)* |
| *Clinical routine* | *Development (cross-validation)* | *0.86*  *(0.84; 0.88)* | *0.88 (0.87; 0.88)* | *0.11 (0.10; 0.12)* | ***0.84 (0.80; 0.89)*** | *0.88 (0.87; 0.89)* | *0.13 (0.12; 0.14)* | *1.00 (0.99; 1.00)* |
| *Amylo-Detect* | *Independent internal validation* | ***0.91 (0.88; 0.94)*** | ***0.93 (0.92; 0.94)*** | ***0.54 (0.45; 0.63)*** | *0.64 (0.55; 0.74)* | ***0.94 (0.93; 0.95)*** | ***0.32 (0.28; 0.37)*** | *0.98 (0.98; 0.99)* |
| *Clinical routine* | *Independent internal validation* | *0.84 (0.80; 0.87)* | *0.79 (0.78; 0.81)* | ***0.14 (0.12; 0.16)*** | ***0.88 (0.81; 0.94)*** | *0.79 (0.77; 0.81)* | *0.15 (0.14; 0.17)* | ***0.99 (0.99; 1.00)*** |
| *Amylo-Detect* | *External validation* | ***0.91***  ***(0.89; 0.93)*** | ***0.87 (0.85; 0.89)*** | ***0.56 (0.48; 0.63)*** | *0.73 (0.66; 0.80)* | ***0.88 (0.87; 0.90)*** | ***0.39 (0.35; 0.44)*** | *0.97 (0.96; 0.98)* |
| *Clinical routine* | *External validation* | *0.85*  *(0.83; 0.87)* | *0.75 (0.73; 0.77)* | ***0.27 (0.25; 0.29)*** | ***0.97 (0.94; 0.99)*** | *0.73 (0.70; 0.75)* | *0.27 (0.26; 0.29)* | ***1.00 (0.99; 1.00)*** |

**Comment 2.6: Prognostic analyses are interesting but currently overinterpreted**

**The association between model-positive status and all-cause mortality or heart failure hospitalisation is clinically interesting. However, the Cox models appear to adjust only for history of cancer. This is not sufficient to establish independent prognostic value, given that model predictions are likely associated with age, sex, atrial fibrillation, renal dysfunction, heart failure history, echocardiographic abnormalities, and other comorbidities.**

**The prognostic analysis begins at the time of scintigraphy and does not demonstrate that using Amylo-Detect would shorten diagnostic delay, alter treatment decisions, or improve outcomes.**

**Suggested revision: Claims around improved outcomes should be softened. The authors should either adjust for clinically relevant covariates or clearly label the prognostic analysis as exploratory and hypothesis-generating. Suggested covariates include age, sex, renal function, atrial fibrillation, heart failure history, relevant echocardiographic variables, and scintigraphy result.**

**Response:** Thank you for this suggestion. We have re-run the Cox models adjusting for cancer, age, sex, BMI, renal insufficiency, atrial fibrillation, history of heart failure, COPD, hypertension, diabetes, coronary artery disease, and carpal tunnel syndrome. Amylo-Detect remained independently prognostic for both clinical endpoints, all-cause mortality and heart failure hospitalization.

***Figure 4.*** *Time-to-event analysis results after multivariate adjustment (cancer, age, sex, BMI, renal insufficiency, atrial fibrillation, hypertension, diabetes, coronary artery disease, COPD, carpal tunnel syndrome, heart-failure history, and interventricular septum thickness) for all-cause mortality and heart failure hospitalization.*

Since scintigraphy referral recommendation is the prediction target, the model was trained on (and the primary clinical use scenario), we did not include scintigraphy results in the model as Amylo-Detect should be employed before scintigraphy.

We adapted the Methods section to include the multivariate adjustments and added a paragraph to the Results highlighting Amylo-Detect as independently prognostic.

*In addition, separate Cox proportional hazard models were adjusted for cancer, age, sex, BMI, renal insufficiency, atrial fibrillation, hypertension, diabetes, coronary artery disease, COPD, carpal tunnel syndrome, heart-failure history, and interventricular septum thickness. A positive Amylo-Detect prediction remained independently associated with all-cause mortality (development: adjHR 1.39 [95% CI1.19; 1.62]; validation: adjHR 1.83 [95% CI 1.19; 2.83]) and HFH (development adjHR 1.39 ]95% CI 1.16; 1.67]; validation adjHR 1.5 [95% CI1.04; 2.16]) (Figure 4).*

**Comment 2.7: The public web app is promising, but implementation safety needs improvement**

**I reviewed the publicly available Amylo-Detect web application. The interface is intuitive, easy to use, and represents a useful strength of the manuscript. The public-facing tool improves accessibility and may facilitate external validation.**

**However, I noted two usability and clinical safety issues:**

**First, the risk output at the bottom of the app appears to remain green regardless of whether the predicted risk is low or high. Given that the tool is intended to support clinical risk assessment, the output should either be neutral in colour or clearly risk-stratified. A uniformly green output may unintentionally reassure users even when the predicted risk is high.**

**Second, submitting the form without entering any parameters still generates a predicted risk of 4.41%. This presumably reflects the model’s imputation pipeline treating all variables as missing and producing a baseline imputed estimate. However, from a clinical usability perspective, this is potentially confusing, even with the "take inference with caution" warning. A user may interpret this as a meaningful patient-specific prediction despite providing no patient-specific information.**

**Suggested revision: The app should prevent submission when no parameters are entered, or clearly label such outputs as not clinically interpretable. The app should specify the absolute minimum input requirements for meaningful use (I note there is a "mandatory parameters" section) and provide clear warnings when predictions are based on extensive missingness. The risk display should be colour coded according to predefined risk categories, or alternatively use neutral formatting with clear explanatory text. The app should also include an intended-use statement, including that the model predicts CA-suggestive scintigraphic uptake rather than confirmed CA, and that it does not replace clinical assessment, monoclonal protein testing, or confirmatory imaging.**

**Response:** We have updated the public web app. Empty submissions no longer generate a prediction and are flagged as not clinically interpretable. The output is now risk-stratified with color coding tied to predefined probability bands rather than uniformly green. The minimum input requirement (at least one valid value, with all mandatory parameters recommended) is now stated in the app, and a warning is shown when predictions rely on extensive missingness (fewer than 6 of 11 parameters provided), in addition to the existing warning for incomplete mandatory fields. An intended-use statement was added, specifying that the model predicts cardiac amyloidosis-suggestive scintigraphy uptake rather than confirmed cardiac amyloidosis, and that it does not replace clinical assessment, monoclonal protein testing or confirmatory imaging. An example output of the manual mode is shown below.

**Comment 2.8: Open science, reproducibility, and software transparency need strengthening**

**PLOS Digital Health asks reviewers to assess whether the study has sufficient methodological detail, whether software is freely available, and whether the work follows appropriate open science standards.**

**The manuscript should provide sufficient information for independent researchers to reproduce model inference. The data availability statement also appears inconsistent between the submission metadata and the manuscript text. One section states that data may be shared upon reasonable request with ethical approval, while the manuscript states that data cannot be shared due to ethical restrictions.**

**Suggested revision: The authors should provide the final selected variables, model coefficients if logistic regression-based, preprocessing pipeline, imputation method, hyperparameters, feature definitions, and versioned source code where possible. If patient-level data cannot be shared, the authors should provide synthetic/example input data and a reproducible inference script. The data availability statement should be harmonised.**

**Response:** We thank the reviewer for the suggestion. We now explicitly provide the final selected features in the Results section.

*A total of 11 features were selected into the final model, including carpal tunnel syndrome, atrial fibrillation, sex, left ventricular diastolic dysfunction, interventricular septum (IVS) thickness and atrial diameter on echocardiography, age, blood urea nitrogen (BUN), history of heart failure, NT-proBNP, and triglycerides (Supplemental Table S4).*

We now include the regression coefficients in **Supplemental Table S4** and added a corresponding reference in the Results section.

***Table S4.*** *Coefficients of the logistic regression model.*

| ***Feature*** | ***Coefficient*** | ***Definition*** |
| --- | --- | --- |
| *Carpal tunnel syndrome* | *5.11* | *Either ICD-10 code G56 or electrophysiological examination* |
| *Atrial fibrillation* | *1.28* | *ICD-10 I48* |
| *Male sex* | *1.02* | *Based on patient record* |
| *Left ventricular function (diastolic)* | *0.58* | *Based on echocardiographic reports; grade 1: ejection fraction (EF) ≥50%; grade 2: EF 49-41%; grade 3: EF 40-31%; grade 4: EF <31%* |
| *IVS thickness* | *0.31* | *Based on echocardiographic reports; in mm* |
| *Age* | *0.16* | *Based on patient record* |
| *BUN* | *0.04* | *Based on laboratory blood analysis (mg/dL)* |
| *History of heart failure* | *0.02* | *Any heart failure diagnosis (ICD-10 I50) in patient records* |
| *proBNP* | *0.01* | *Based on laboratory blood analysis (pg/mL)* |
| *Triglycerides* | *-0.01* | *Based on laboratory blood analysis (mg/dL)* |
| *Right atrial diameter* | *-0.07* | *Based on echocardiographic reports; in mm* |
| *Intercept* | *-17.39* |  |

The model is available for full inference, including the preprocessing and imputation pipeline via the web app. This allows for fast reproducibility and easy use, which we believe is particularly important for clinical users. We make the web app freely available for both single-patient inference and batch inference (for research). Sharing of patient data is not permitted by the ethics approval of this study. We now provide a synthetic example dataset that can be loaded via an additional interface section in the batch inference interface of the Amylo-Detect web application. In addition, we added a section for the manual input mode of the web app that allows for automatic filling of features with realistic example values. The newly introduced interface section provides the user with separate options for loading an example with cardiac amyloidosis, likely and unlikely. A reworked example for both the manual and batch input interface are shown below.

Manual input:

Batch input:

**Comment 2.9: Claims around novelty and generalisability should be more balanced**

**The study is larger than many prior amyloidosis AI studies and includes external validation, which are important strengths. However, the manuscript should avoid describing the cohort as "unbiased" without qualification. These are all-comer scintigraphy referrals, not all-comer hospital patients.**

**The discussion should more clearly distinguish between three possible use cases:**

**1.Risk stratification among patients already referred for scintigraphy.**

**2.Opportunistic identification among patients undergoing scintigraphy for non-cardiac indications.**

**3.Broader automated EHR screening across hospital populations.**

**The current data most strongly support the first two, but not yet the third.**

**Response:**

We thank the reviewer for the comment and agree that ‘unbiased’ in our statement is misleading and we have therefore removed the word ‘unbiased’ from our statement. Our statement that the cohort consists of ‘unbiased all-comer referrals for bone scintigraphy’ was referring to the fact that we used a maximally inclusive real-world population that underwent bone scintigraphy without any artificial inclusion or exclusion criteria restricting applicability in this population.

We further added the three-use-case distinction and state that our data support risk stratification among patients referred for scintigraphy and opportunistic identification among patients scanned for non-cardiac indications, but not yet broader hospital-wide EHR screening. Additionally, while the model may be used for clinical purposes, we believe it has important implications for clinical research, providing the possibility to identify previously missed patients with cardiac-amyloidosis suggestive uptake. This use-case is particularly important since cardiac amyloidosis is considered underdiagnosed and much of the research regarding cardiac amyloidosis is currently targeted towards identification and markers related to previously undetected cardiac amyloidosis patients. We therefore also added this, third, supported use case.

*Our results support three use cases of Amylo-Detect. First, risk stratification among patients already referred for scintigraphy. Second, opportunistic identification among patients undergoing scintigraphy for non-cardiac indications. Third, identification of previously missed cases for study cohorts.*

We removed any statements indicating that the model has been trained or validated or can be used for screening a hospital-wide population and added an explicit statement to clarify.

*We show that Amylo-Detect can identify patients at risk of cardiac-amyloidosis-suggestive scintigraphic uptake among patients whose clinical features resemble those of a bone-scintigraphy-referral population. Although the study population spanned a wide range of clinical indications beyond suspected CA and a wide range of ages and comorbidities, this study was neither developed nor validated in an unselected hospital population. Broader hospital-wide screening requires prospective validation with systematic assessment of CA in all patients.*

**Comment 2.10: Potential conflicts of interest should be contextualised**

**The authors disclose several relevant relationships with pharmaceutical companies active in cardiac amyloidosis and one author’s role as co-founder of a company developing ECG algorithms for amyloidosis. The disclosures are appropriately listed, but the manuscript would benefit from a clear statement that these entities had no role in study design, data collection, analysis, interpretation, software deployment, or manuscript preparation, where applicable.**

**Response:** We have added the following statement to the Disclosures.

*The companies and organizations listed above had no role in the study design, data collection, analysis, interpretation, software development and deployment or manuscript preparation. The companies did not provide funding for this study.*

**Minor comments**

**Comment 2.11: The temporal split between the development and internal validation cohorts should be clarified. The abstract states that patients referred before August 2020 formed the development cohort, whereas the Methods appear to describe patients examined before July 2020 as the development cohort. Please harmonise the dates and specify the exact cut-off date, including whether patients examined during July 2020 were included in development or internal validation.**

**Response:** The cut-off is harmonized to August 2020 throughout. Patients referred before August 2020 (up to and including July 2020) form the development cohort. The remainder form the internal validation cohort. The Methods text, stating before July 2020, has been corrected.

**Comment 2.12: Please clearly define "high-grade cardiac uptake on scintigraphy" in your manuscript methods section - I assume this is Perugini grade ≥2. Was this based on planar images, clinical report, expert adjudication? Was the same method used across both Vienna and Essen?**

**Response:** We have added a section ‘Image acquisition and annotation’ to the Methods section, including a full grading description and a specification regarding high-grade uptake (Perugini grade ≥2).

*Cardiac tracer uptake was annotated according to the Perugini classification*^2,3^*, whereby grade 0 represents no cardiac uptake, grade 1 minor cardiac uptake (heart less than bone) and grade 2 or 3 intense cardiac uptake (heart equal to or greater than bone). Grade 2 or higher uptake with absence is highly specific and sensitive for cardiac amyloidosis. Grading was therefore dichotomised into high-grade (cardiac-amyloidosis-suggestive uptake: grade 2 or 3) and non-suggestive uptake (grade 0 or 1). For the Vienna cohorts, grading was performed by a centralized core laboratory at the Medical University of Vienna comprising three independent, mutually blinded specialists with at least 5 years of experience (CN, TTW and JN). Discrepancies, which occurred almost exclusively at the grade 1 versus grade 2 border, were resolved by consensus reading by two further specialists with at least 10 years of experience (RC and Alexander R. Haug). At the external site, Perugini grades were annotated based on the clinical routine imaging reports and stratified into high-grade (Perugini grade ≥2) or non-suggestive uptake (Perugini grade ≤1).*

**Comment 2.13: Please report AUPRC in addition to AUC because the outcome prevalence is low.**

**Response:** AUPRC was 0.31 (95% CI 0.237 to 0.389), 0.54 (95% CI 0.446 to 0.631) and 0.56 (95% CI 0.480 to 0.634) in the development, independent internal validation and external validation cohorts for the Amylo-Detect, which substantially outperformed clinical routine referral with AUPRCs of 0.11 (95% CI 0.102 to 0.124), 0.14 (0.123 to 0.158) and 0.27 (0.250 to 0.289) in all cohorts. We added AUPRC for all cohorts to Table 1.

***Table 1.*** *Machine learning performance for the prediction of cardiac amyloidosis-indicative uptake on bone scintigraphy.*

| *Approach* | *Cohort* | *AUC*  *(95% CI)* | *ACC (95% CI)* | *AUPRC (95% CI)* | *SNS*  *(95% CI)* | *SPC*  *(95% CI)* | *PPV*  *(95% CI)* | *NPV*  *(95% CI)* |
| --- | --- | --- | --- | --- | --- | --- | --- | --- |
| *Amylo-Detect* | *Development (cross-validation)* | ***0.93***  ***(0.91; 0.95)*** | ***0.93 (0.92; 0.93)*** | ***0.31 (0.24; 0.39)*** | *0.71 (0.63; 0.79)* | ***0.93 (0.92; 0.93)*** | ***0.14 (0.12; 0.15)*** | *1.00 (0.99; 1.00)* |
| *Clinical routine* | *Development (cross-validation)* | *0.86*  *(0.84; 0.88)* | *0.88 (0.87; 0.88)* | *0.11 (0.10; 0.12)* | ***0.84 (0.80; 0.89)*** | *0.88 (0.87; 0.89)* | *0.13 (0.12; 0.14)* | *1.00 (0.99; 1.00)* |
| *Amylo-Detect* | *Independent internal validation* | ***0.91 (0.88; 0.94)*** | ***0.93 (0.92; 0.94)*** | ***0.54 (0.45; 0.63)*** | *0.64 (0.55; 0.74)* | ***0.94 (0.93; 0.95)*** | ***0.32 (0.28; 0.37)*** | *0.98 (0.98; 0.99)* |
| *Clinical routine* | *Independent internal validation* | *0.84 (0.80; 0.87)* | *0.79 (0.78; 0.81)* | ***0.14 (0.12; 0.16)*** | ***0.88 (0.81; 0.94)*** | *0.79 (0.77; 0.81)* | *0.15 (0.14; 0.17)* | ***0.99 (0.99; 1.00)*** |
| *Amylo-Detect* | *External validation* | ***0.91***  ***(0.89; 0.93)*** | ***0.87 (0.85; 0.89)*** | ***0.56 (0.48; 0.63)*** | *0.73 (0.66; 0.80)* | ***0.88 (0.87; 0.90)*** | ***0.39 (0.35; 0.44)*** | *0.97 (0.96; 0.98)* |
| *Clinical routine* | *External validation* | *0.85*  *(0.83; 0.87)* | *0.75 (0.73; 0.77)* | ***0.27 (0.25; 0.29)*** | ***0.97 (0.94; 0.99)*** | *0.73 (0.70; 0.75)* | *0.27 (0.26; 0.29)* | ***1.00 (0.99; 1.00)*** |

**Comment 2.14: Please provide confusion matrices for each validation cohort at the selected threshold.**

**Response:** Confusion matrices at the 0.043 (recalibrated from 0.77) threshold are added for all cohorts as a new supplementary Figure. We have added a reference in the Results section of the ‘Diagnostic performance of the machine learning model’ section of the Results.

***Figure S5****. Confusion matrices at threshold 0.043 for the cross-validation development (A), temporal internal validation (B) and external validation (C).*

**Comment 2.15: Please clarify how the Mayo ATTR-CM score was calculated when variables were missing.**

**Response:** To ensure comparability, imputation of missing values required for calculation of the Mayo ATTR-CM score was performed using the same k-nearest-neighbors imputation procedure used for the machine learning models. We added a corresponding statement to the Methods section.

*To ensure comparability, imputation of missing values required for the calculation of the Mayo ATTR-CM score was performed using the same k-nearest-neighbors imputation procedure used for the machine learning models.*

**Comment 2.16: Please clarify whether the external validation site used the same DPD acquisition, reconstruction, reporting, and grading protocols as the development site.**

**Response:** For all cohorts, whole-body imaging were performed according to standardized institutional routine protocols with image acquisition at least 2 hours after ^99m^Tc-DPD injection in accordance with clinical guidelines. Scanners differed between and within cohorts but are not expected to influence diagnostic image reading. Diagnostic image reading was performed using the Perugini grading scheme with the Vienna data being more comprehensively annotated by consensus reading while the annotations of the Essen cohort were derived based on the final grading performed during clinical routine. While we expect that the reading approach in the Vienna cohorts is more precise, clinical routine procedures are generally accepted as accurate and no consensus reading is performed or recommended in clinical routine. We added a corresponding section ‘Image acquisition and annotation’.

*Image acquisition was performed using standardized institutional routine protocols for ^99m^Tc-DPD whole-body scintigraphy with post-injection acquisition times of ≥2 hours in all cohorts. Cardiac tracer uptake was annotated according to the Perugini classification*^2,3^*, whereby grade 0 represents no cardiac uptake, grade 1 minor cardiac uptake (heart less than bone) and grade 2 or 3 intense cardiac uptake (heart equal to or greater than bone). Grade 2 or higher uptake with absence is highly specific and sensitive for cardiac amyloidosis. Grading was therefore dichotomised into high-grade (cardiac-amyloidosis-suggestive uptake: grade 2 or 3) and non-suggestive uptake (grade 0 or 1). For the Vienna cohorts, grading was performed by a centralized core laboratory at the Medical University of Vienna comprising three independent, mutually blinded specialists with at least 5 years of experience (CN, TTW and JN). Discrepancies, which occurred almost exclusively at the grade 1 versus grade 2 border, were resolved by consensus reading by two further specialists with at least 10 years of experience (RC and Alexander R. Haug). At the external site, Perugini grades were annotated based on the clinical routine imaging reports and stratified into high-grade (Perugini grade ≥2) or non-suggestive uptake (Perugini grade ≤1).*

**Comment 2.17: Please avoid wording such as "life-saving treatments" unless specifically justified, as it may sound promotional, suggest "guideline-directed".**

**Response:** Replaced. The abstract now reads *guideline-directed disease-modifying treatments*. We would still like to highlight that these treatments are indeed life-saving. They have been approved through the application of large-scale international multi-center randomized controlled clinical phase 3 trials and have been approved and successfully applied in clinical routine since 2019 in the US and since 2020 in the EU.

**Comment 2.18: The phrase "promises to address diagnostic delays and improve outcomes" should be softened to "may help address diagnostic delays, pending prospective evaluation".**

**Response:** Revised to *may help address diagnostic delays, pending prospective evaluation* in the abstract's conclusion.

**Comment 2.19: Please correct typographical errors, including "cardac" and inconsistent capitalisation/formatting of HFpEF.**

**Response:** Corrected cardac to cardiac and standardized HFpEF throughout.

**Comment 2.20: Please provide more detail on the feature selection process, including the exact final 13 variables used in the model.**

**Response:** Feature selection was performed using the minimum redundancy maximum relevance (mRMR) algorithm^4^. A total of log2(n) features were selected, where n represents the number of instances in the development set. Additionally, we removed features from the selected feature set if their normalized logistic regression coefficients were less than 0.01 to avoid features with low incremental value. This reduced the number of features from 13 to 11, which were used in the final model. We added a corresponding statement to the Methods section and corrected the number of final features mentioned in the Results section from 13 to 11.

*For feature selection, a total of log2(n) features were selected were n represented the number of total patients in the development cohort. Additionally, features were removed if their normalized logistic regression coefficients were below 0.01 to avoid features with minimal effect on the model.*

We further provide a statement explicitly mentioning the number and name of features included in the final model. *A total of 11 features were selected into the final model, including carpal tunnel syndrome, atrial fibrillation, sex, left ventricular diastolic dysfunction, interventricular septum (IVS) on echocardiography, age, blood urea nitrogen (BUN), history of heart failure, NT-proBNP, triglycerides and atrial diameter on echocardiography.*

**Comment 2.21: Please include a clearer explanation of how the app handles missing values, including whether a fully missing input vector is allowed by design.**

**Response:** The app imputes missing values using the same kNN imputation model used for all analyses in the publication. We updated the web app to not accept a fully missing input vector (all parameters missing) and instead show a warning with a red underlay that clearly states that features are missing and no result can be computed. For feature vectors with at least one filled parameter, the model will compute a result. If not all mandatory features are filled, the app will provide a warning with a yellow underlay stating that not all mandatory features were filled in and that results have to be taken with caution. We intentionally retain the user's ability to generate results with low numbers of available parameters and leave the decision to the user whether the results can be used for their purpose.

**Comment 2.22: Please ensure ATTR-CM or CM is defined in full at first use.**

**Response:** Transthyretin amyloid cardiomyopathy (ATTR-CM) is now defined at first use and used consistently thereafter.

# **Additional corrections made for internal consistency**

During revision, we identified and corrected several internal inconsistencies that we corrected:

**-** Clinically missed cases additionally detected by Amylo-Detect were reported as 12/42 (29%) in the abstract but 10/38 (26%) in the Results. These are now reconciled to a single value, 12/42 (29%).

- In the Discussion we once referenced the development cross-validation AUC to be 0.94 instead of 0.93.

# **Journal requirements**

**1. Please send a completed 'Competing Interests' statement, including any COIs declared by your co-authors. If you have no competing interests to declare, please state "The authors have declared that no competing interests exist". Otherwise please declare all competing interests beginning with the statement "I have read the journal's policy and the authors of this manuscript have the following competing interests:"**

We now include a Competing Interests statement, which is also part of the manuscript. CN reports speaker/consulting honoraria from Pfizer, Bayer, Prothena, and Böhringer Ingelheim and research contracts with Pfizer, AstraZeneca, the Austrian Society of Cardiology, the European Association of Cardiovascular Imaging, and the Austrian Science Fund. CPS declares consulting and/or speaker fees from Pfizer, Hermes Medical Solutions, CMI Experts, the European School of Multimodality Imaging and Therapy (ESMIT), and the European Society of Radiology. CPS receives funding from the FWF Austrian Science Fund (10.55776/J4998). MH has received lecture fees from Siemens Healthineers and GE Healthcare. MH has received consulting fees from Evomics. TR has received honoraria, lecture fees, and grant support from Edwards Lifesciences, AstraZeneca, Bayer, Novartis, Berlin Chemie, Daiicho-Sankyo, Boehringer Ingelheim, Novo Nordisk, Cardiac Dimensions, and Pfizer. TR is co-founder of mycor GmbH, a company focusing on the development of ECG-algorithms for the identification of amyloidosis. The remaining authors have nothing to disclose.

The companies and organizations listed above had no role in the study design, data collection, analysis, interpretation, software development and deployment or manuscript preparation. The companies did not provide funding for this study.

ChatGPT and Grammarly were used to refine the phrasing of the manuscript; all scientific content and conclusions are the authors’ own.

**2. For studies involving third-party data, we encourage authors to share any data specific to their analyses that they can legally distribute. PLOS recognizes, however, that authors may be using third-party data they do not have the rights to share. When third-party data cannot be publicly shared, authors must provide all information necessary for interested researchers to apply to gain access to the data. (https://journals.plos.org/plosone/s/data-availability#loc-acceptable-data-access-restrictions)**

The study includes data from the General Hospital Vienna and the University Clinics Essen. Sharing of data is not permitted by the ethical approval of this study. If readers are interested in obtaining the study employed in this study, they may contact the corresponding author, who will provide support in contacting the relevant authorities to obtain the data if an ethics approval is available. We include a corresponding statement in our manuscript.

*The data supporting this study cannot be shared due to restrictions imposed by the ethical approvals. Researchers interested in accessing the data may contact the corresponding author to discuss potential options.*

**For any third-party data that the authors cannot legally distribute, they should include the following information in their Data Availability Statement upon submission:**

**1) A description of the data set and the third-party source**

**2) If applicable, verification of permission to use the data set**

**3) Confirmation of whether the authors received any special privileges in accessing the data that other researchers would not have**

**4) All necessary contact information others would need to apply to gain access to the data**

We added a corresponding statement to the Data availability statement in the manuscript.

*Data was obtained from the patient records, imaging archives of the Vienna General Hospital and the University Clinics Essen, as well as national databases, including the Austrian Death Registry, the Vienna-Health-Association database, and the Austrian nationwide electronic health records.*

*In order to obtain the data, please contact the corresponding author, who will mediate with the relevant authorities depending on the type of required data (hospital, university, ethics committee data clearance office, and legal department).*

.

**3. Some material included in your submission may be copyrighted. According to PLOS’s copyright policy, authors who use figures or other material (e.g., graphics, clipart, maps) from another author or copyright holder must demonstrate or obtain permission to publish this material under the Creative Commons Attribution 4.0 International (CC BY 4.0) License used by PLOS journals. Please closely review the details of PLOS’s copyright requirements here: PLOS Licenses and Copyright. If you need to request permissions from a copyright holder, you may use PLOS's Copyright Content Permission form.**

**Please respond directly to this email or email the journal office and provide any known details concerning your material's license terms and permissions required for reuse, even if you have not yet obtained copyright permissions or are unsure of your material's copyright compatibility.**

**Potential Copyright Issues:**

**a. Figures 1 and 2: Please confirm whether you drew the images / clip-art within the figure panels by hand. If you did not draw the images, please provide (a) a link to the source of the images or icons and their license / terms of use; or (b) written permission from the copyright holder to publish the images or icons under our CC-BY 4.0 license. Alternatively, you may replace the images with open source alternatives. See these open source resources you may use to replace images / clip-art:
- https://commons.wikimedia.org
-** [**https://openclipart.org/**](https://openclipart.org/)

All figures were created by the authors or explicitly acknowledged individuals. Figures 1 and 2 were created using Biorender and are appropriately acknowledged. We attached the corresponding publication licenses to the submission.

**4. Please upload separate figure files in .tif or .eps format. Also, remove the figures from your manuscript file but keep the legends.**

**For more information about figure files please see our guidelines:**

**https://journals.plos.org/digitalhealth/s/figures**

**https://journals.plos.org/digitalhealth/s/figures#loc-file-requirements**

Done

**5. We have noticed that you have uploaded Supporting Information files, but you have not included a list of legends. Please add a full list of legends for your Supporting Information files after the references list.**

We added a list of legends after the reference list in the Supporting Information files.

**References**

1.	Writing Committee *et al.* Transthyretin cardiac amyloidosis evaluation and management: 2025 ACC Concise Clinical Guidance. *J. Am. Coll. Cardiol.* (2025) doi:10.1016/j.jacc.2025.09.004.

2.	Perugini, E. *et al.* Noninvasive etiologic diagnosis of cardiac amyloidosis using 99mTc-3,3-diphosphono-1,2-propanodicarboxylic acid scintigraphy. *J. Am. Coll. Cardiol.* **46**, 1076–1084 (2005).

3.	Gillmore, J. D. *et al.* Nonbiopsy Diagnosis of Cardiac Transthyretin Amyloidosis. *Circulation* **133**, 2404–2412 (2016).

4.	Peng, H., Long, F. & Ding, C. Feature selection based on mutual information: criteria of max-dependency, max-relevance, and min-redundancy. *IEEE Trans. Pattern Anal. Mach. Intell.* **27**, 1226–1238 (2005).
