# Supplementary material for: Screening for patients at risk for cardiac amyloidosis via electronic health records: A multicenter machine learning development and validation study
Source: PLOS Digit Health. 2026 Oct 8;5(10):e0001637. doi: 10.1371/journal.pdig.0001637 (PMC13649026; doi:10.1371/journal.pdig.0001637)
Supplement: Peer review history

# Peer review: Decision Letter 0

**DOI:** 10.1371/journal.pdig.0001637.r001

**Authors:** Peter H Charlton

26 May 2026

Response to Reviewers Revised Manuscript with Track Changes Manuscript **Journal Requirements:**

1. Please send a completed 'Competing Interests' statement, including any COIs declared by your co-authors. If you have no competing interests to declare, please state "The authors have declared that no competing interests exist". Otherwise please declare all competing interests beginning with the statement "I have read the journal's policy and the authors of this manuscript have the following competing interests:"

2. For studies involving third-party data, we encourage authors to share any data specific to their analyses that they can legally distribute. PLOS recognizes, however, that authors may be using third-party data they do not have the rights to share. When third-party data cannot be publicly shared, authors must provide all information necessary for interested researchers to apply to gain access to the data. (https://journals.plos.org/plosone/s/data-availability#loc-acceptable-data-access-restrictions)

For any third-party data that the authors cannot legally distribute, they should include the following information in their Data Availability Statement upon submission:

1) A description of the data set and the third-party source

2) If applicable, verification of permission to use the data set

3) Confirmation of whether the authors received any special privileges in accessing the data that other researchers would not have

4) All necessary contact information others would need to apply to gain access to the data

3. Some material included in your submission may be copyrighted. According to PLOS’s copyright policy, authors who use figures or other material (e.g., graphics, clipart, maps) from another author or copyright holder must demonstrate or obtain permission to publish this material under the Creative Commons Attribution 4.0 International (CC BY 4.0) License used by PLOS journals. Please closely review the details of PLOS’s copyright requirements here: PLOS Licenses and Copyright. If you need to request permissions from a copyright holder, you may use PLOS's Copyright Content Permission form.

Please respond directly to this email or email the journal office and provide any known details concerning your material's license terms and permissions required for reuse, even if you have not yet obtained copyright permissions or are unsure of your material's copyright compatibility.

Potential Copyright Issues:

a. Figures 1 and 2: Please confirm whether you drew the images / clip-art within the figure panels by hand. If you did not draw the images, please provide (a) a link to the source of the images or icons and their license / terms of use; or (b) written permission from the copyright holder to publish the images or icons under our CC-BY 4.0 license. Alternatively, you may replace the images with open source alternatives. See these open source resources you may use to replace images / clip-art:

- https://commons.wikimedia.org

- https://openclipart.org/

4. Please upload separate figure files in .tif or .eps format. Also, remove the figures from your manuscript file but keep the legends.

For more information about figure files please see our guidelines:

https://journals.plos.org/digitalhealth/s/figures

https://journals.plos.org/digitalhealth/s/figures#loc-file-requirements

5. We have noticed that you have uploaded Supporting Information files, but you have not included a list of legends. Please add a full list of legends for your Supporting Information files after the references list.

**Additional Editor Comments (if provided):** **Reviewers' Comments:**

**Comments to the Author**

1. Does this manuscript meet PLOS Digital Health’s publication criteria?

Reviewer #1: Yes

Reviewer #2: Partly

**********

2. Has the statistical analysis been performed appropriately and rigorously?

Reviewer #1: Yes

Reviewer #2: No

**********

3. Have the authors made all data underlying the findings in their manuscript fully available (please refer to the Data Availability Statement at the start of the manuscript PDF file)?

The PLOS Data policy

Reviewer #1: Yes

Reviewer #2: No

**********

4. Is the manuscript presented in an intelligible fashion and written in standard English?

Reviewer #1: Yes

Reviewer #2: Yes

**********

**Reviewer #1:**  In this study, the authors use a trained machine learning algorithm to detect patients with cardiac amyloidosis from electronic health records. Treatment of cardiac amyloidosis has developed rapidly in recent years with nuclear imaging and new medical interventions. Still, the main issue is early disease detection to enable timely referral of patients to diagnostic imaging and therapy. Therefore, this is clinically meaningful and important field of research. I have some further comments below.

Authors have developed an early detection algorithm for cardiac ATTR amyloidosis (Perugini grade 2 or 3). However, the early ATTR might present as Perugini grade 1 uptake on scintigraphy. This will impact the algorithm's training, since the earliest disease may be missed. Positive algorithm findings might be classified as false positives due to less than grade 2 uptake.

Patients with cardiac amyloidosis frequently have multiple health care contacts prior to the correct diagnosis, and the diagnostic delay is long. This is an opportunity for the algorithm to detect the disease (there should be lots of data in positive cases). I would emphasise that there is frequently a lot of EHR data available prior to the correct diagnosis (for example, study: https://doi.org/10.1161/CIRCULATIONAHA.118.038169)

The median age of the development cohort was (64 y, 52-73 y). This is quite a low age range for ATTR cardiomyopathy patients. Does this affect the algorithm's training? Since older patients' electronic health record data might differ from that of younger patients? How common is the hereditary form in Austria among younger patients?

The authors developed a model with a high AUC. Another approach would be to develop a model with very high sensitivity to detect all patients with an elevated risk of ATTR. Further screening with SPECT is quite affordable. This would increase the yield of the ATTR detection but would demand more resources for follow-up nuclear imaging. Is this approach be possible from a practical / economic point of view?

The algorithm relies on the electronic health record data. In health care systems with poor communication between hospitals or problems integrating primary healthcare and cardiology clinic data, this kind of algorithms value might be limited. Could the authors comment on how this approach would work, for example, in the United States, in a healthcare setting with multiple private healthcare providers?

Another limitation is the use of echocardiography data in the algorithm. Would the patient need a prior cardiology clinic visit to be detected by this algorithm?

Could authors characterize the features of patients detected by Amylo-Detect and missed by the clinical standard? Was there some clinician bias to age, gender or was it more frequently early disease harder to detect?

**Reviewer #2:**  Summary

This manuscript presents Amylo-Detect, an ML model designed to identify patients at risk of CA using routinely available structured EHR data. The model was developed in a large retrospective cohort of consecutive patients referred for DPD bone scintigraphy at Vienna General Hospital and validated in both a temporally separated internal cohort and an external cohort from University Hospital Essen. The model predicts high-grade cardiac uptake on scintigraphy, defined as Perugini grade ≥2, as a marker suggestive of CA.

The study includes 9,170 patients in the development cohort, 2,446 in the internal validation cohort, and 1,521 in the external validation cohort. High-grade cardiac uptake was present in 388 patients overall. Amylo-Detect achieved strong discrimination, with AUCs of 0.93 in development, 0.91 in internal validation, and 0.91 in external validation. The authors report that the model outperformed an existing Mayo ATTR-CM score and clinical referral indication in several metrics. They also report that model-positive status was associated with all-cause mortality and heart failure hospitalisation.

The manuscript addresses an important clinical problem. CA remains underdiagnosed, and timely identification of patients suitable for confirmatory testing is a major clinical bottleneck. The study has several strengths, including the large sample size, temporal internal validation, external validation, use of structured EHR data, retention of patients with missing data, comparison with existing approaches, and public availability of a web application.

However, several issues need to be addressed before the conclusions are fully supported. Most importantly, the model was developed and validated only in patients already referred for bone scintigraphy, yet parts of the manuscript imply applicability to broader hospital-wide EHR screening. The primary outcome is CA-suggestive uptake rather than confirmed CA. The clinical utility of the selected threshold is not yet adequately demonstrated, especially given lower sensitivity than clinical referral indication. Further clarity is also needed around missing data handling, calibration, threshold selection, external validation using limited variables, and safe implementation of the public web app.

---

Major comments

1. The intended-use population is broader than the evidence supports

The manuscript frames Amylo-Detect as a tool for identifying patients at risk of CA using EHR data. However, all development and validation cohorts consisted of patients who had already been referred for DPD bone scintigraphy. This is a highly selected population. Even if the cohort includes "all-comer referrals" for scintigraphy, it does not represent an unselected hospital, cardiology, heart failure, aortic stenosis, primary care, or general EHR population.

This distinction is central to the clinical interpretation of the work. A model that performs well among patients already selected for scintigraphy may not perform similarly if deployed across all hospital patients. The statement that the results apply to "all patients visiting a hospital" is therefore not supported by the current study design.

Suggested revision: The authors should define the intended-use population more narrowly throughout the manuscript. For example, the model could be framed as identifying patients at risk of CA-suggestive scintigraphy findings among patients with clinical features similar to those entering a scintigraphy-referral population. Alternatively, broader claims would require validation in an unselected EHR population with systematic outcome ascertainment.

2. Clinical utility is not yet adequately demonstrated

The model demonstrates high AUC, but AUC alone is insufficient to establish clinical usefulness for a screening or referral tool. At the selected operating threshold, Amylo-Detect has lower sensitivity than clinical standard in both validation cohorts. In the internal validation cohort, sensitivity is 0.64 for Amylo-Detect vs. 0.88 for clinical standard. In the external validation cohort, sensitivity is 0.73 vs. 0.97.

This is particularly important because the proposed use case is screening or referral support for a progressive and treatable disease. Missing cases may be clinically consequential. The model has higher specificity and PPV than clinical standard, but the trade-off needs to be explicitly justified.

The PPV is also modest, especially in development, where PPV is 0.14. This may be acceptable if the downstream pathway is low burden and cost-effective, but the manuscript does not quantify the number of additional referrals, scans, monoclonal protein tests, false positives, or downstream resource implications.

Suggested revision: The authors should provide decision-curve analysis, net benefit, number needed to screen, number needed to scan, false-positive burden, and threshold-specific performance across clinically plausible thresholds. They should explicitly state whether Amylo-Detect is intended to replace clinical judgement, supplement clinical judgement, or prioritise patients for expert review.

3. The comparison with "clinical standard" needs clearer framing

The comparison with clinical routine is interesting but potentially misleading. "Clinical standard" appears to be inferred from the referral indication among patients who already underwent scintigraphy. This is not the same as evaluating clinician decision-making in an unselected EHR population. Rather, it assesses whether the indication for an already-performed scan included suspected CA.

There is also a conceptual issue with reporting AUC for a binary clinical referral indication and comparing this with a continuous model output. The authors should explain exactly how this AUC was calculated and whether this is a fair comparison.

Suggested revision: The authors should reframe this comparator as "referral indication among scintigraphy recipients" rather than "clinical standard" unless they can provide a more direct comparator to real-world clinician referral decisions.

4. Missing data handling is central and requires more transparency

The manuscript states that patients had a median of 38 missing parameters out of 50, and 6 missing parameters among the 13 selected model parameters. This level of missingness is substantial. The authors appropriately attempt to handle this using imputation, but more detail is required.

The finding that the model performed slightly better in patients with more missing values is potentially reassuring, but it may also suggest that missingness itself is informative. Missingness may encode clinical workflow, investigation availability, institutional practice, or referral patterns rather than disease biology.

External validation was apparently performed using only the six most important parameters. This raises further questions about the true input requirements of the deployed model. It is unclear whether the model requires 50 variables, 13 variables, six variables, or can operate meaningfully with almost no variables.

Suggested revision: Please provide a detailed missingness table by variable and cohort, clarify whether missingness indicators were used, and describe the KNN imputation process in greater detail. The authors should report performance in patients with complete data, patients with only the six key variables, patients without echocardiographic data, and patients without carpal tunnel syndrome coding. They should also discuss whether missingness patterns may encode local clinical workflows.

5. Calibration and threshold selection require stronger reporting

Amylo-Detect is presented as providing a continuous probability score, yet calibration reporting is limited. The manuscript states that risk calibration yielded a Brier score of 0.085 and did not improve with isotonic regression. In a low-prevalence screening setting, this is insufficient to establish that the predicted probabilities are clinically interpretable.

The probability threshold of 0.77 is described as achieving a "reasonable balance" of diagnostic metrics, but this threshold does not appear to be clinically justified. For a screening/referral tool, threshold choice should be linked to intended use, acceptable false-positive burden, acceptable false-negative rate, and downstream testing capacity.

Suggested revision: Please provide calibration plots for all cohorts, calibration slope and intercept, observed versus predicted risk by decile, precision-recall curves, and threshold-specific diagnostic performance tables. Precision-recall analysis is particularly relevant given the low prevalence of the outcome.

6. Prognostic analyses are interesting but currently overinterpreted

The association between model-positive status and all-cause mortality or heart failure hospitalisation is clinically interesting. However, the Cox models appear to adjust only for history of cancer. This is not sufficient to establish independent prognostic value, given that model predictions are likely associated with age, sex, atrial fibrillation, renal dysfunction, heart failure history, echocardiographic abnormalities, and other comorbidities.

The prognostic analysis begins at the time of scintigraphy and does not demonstrate that using Amylo-Detect would shorten diagnostic delay, alter treatment decisions, or improve outcomes.

Suggested revision: Claims around improved outcomes should be softened. The authors should either adjust for clinically relevant covariates or clearly label the prognostic analysis as exploratory and hypothesis-generating. Suggested covariates include age, sex, renal function, atrial fibrillation, heart failure history, relevant echocardiographic variables, and scintigraphy result.

7. The public web app is promising, but implementation safety needs improvement

I reviewed the publicly available Amylo-Detect web application. The interface is intuitive, easy to use, and represents a useful strength of the manuscript. The public-facing tool improves accessibility and may facilitate external validation.

However, I noted two usability and clinical safety issues:

First, the risk output at the bottom of the app appears to remain green regardless of whether the predicted risk is low or high. Given that the tool is intended to support clinical risk assessment, the output should either be neutral in colour or clearly risk-stratified. A uniformly green output may unintentionally reassure users even when the predicted risk is high.

Second, submitting the form without entering any parameters still generates a predicted risk of 4.41%. This presumably reflects the model’s imputation pipeline treating all variables as missing and producing a baseline imputed estimate. However, from a clinical usability perspective, this is potentially confusing, even with the "take inference with caution" warning. A user may interpret this as a meaningful patient-specific prediction despite providing no patient-specific information.

Suggested revision: The app should prevent submission when no parameters are entered, or clearly label such outputs as not clinically interpretable. The app should specify the absolute minimum input requirements for meaningful use (I note there is a "mandatory parameters" section) and provide clear warnings when predictions are based on extensive missingness. The risk display should be colour coded according to predefined risk categories, or alternatively use neutral formatting with clear explanatory text. The app should also include an intended-use statement, including that the model predicts CA-suggestive scintigraphic uptake rather than confirmed CA, and that it does not replace clinical assessment, monoclonal protein testing, or confirmatory imaging.

8. Open science, reproducibility, and software transparency need strengthening

PLOS Digital Health asks reviewers to assess whether the study has sufficient methodological detail, whether software is freely available, and whether the work follows appropriate open science standards.

The manuscript should provide sufficient information for independent researchers to reproduce model inference. The data availability statement also appears inconsistent between the submission metadata and the manuscript text. One section states that data may be shared upon reasonable request with ethical approval, while the manuscript states that data cannot be shared due to ethical restrictions.

Suggested revision: The authors should provide the final selected variables, model coefficients if logistic regression-based, preprocessing pipeline, imputation method, hyperparameters, feature definitions, and versioned source code where possible. If patient-level data cannot be shared, the authors should provide synthetic/example input data and a reproducible inference script. The data availability statement should be harmonised.

9. Claims around novelty and generalisability should be more balanced

The study is larger than many prior amyloidosis AI studies and includes external validation, which are important strengths. However, the manuscript should avoid describing the cohort as "unbiased" without qualification. These are all-comer scintigraphy referrals, not all-comer hospital patients.

The discussion should more clearly distinguish between three possible use cases:

1.Risk stratification among patients already referred for scintigraphy.

2.Opportunistic identification among patients undergoing scintigraphy for non-cardiac indications.

3.Broader automated EHR screening across hospital populations.

The current data most strongly support the first two, but not yet the third.

10. Potential conflicts of interest should be contextualised

The authors disclose several relevant relationships with pharmaceutical companies active in cardiac amyloidosis and one author’s role as co-founder of a company developing ECG algorithms for amyloidosis. The disclosures are appropriately listed, but the manuscript would benefit from a clear statement that these entities had no role in study design, data collection, analysis, interpretation, software deployment, or manuscript preparation, where applicable.

---

Minor comments

1.The temporal split between the development and internal validation cohorts should be clarified. The abstract states that patients referred before August 2020 formed the development cohort, whereas the Methods appear to describe patients examined before July 2020 as the development cohort. Please harmonise the dates and specify the exact cut-off date, including whether patients examined during July 2020 were included in development or internal validation.

2.Please clearly define "high-grade cardiac uptake on scintigraphy" in your manuscript methods section - I assume this is Perugini grade ≥2. Was this based on planar images, clinical report, expert adjudication? Was the same method used across both Vienna and Essen?

3.Please report AUPRC in addition to AUC because the outcome prevalence is low.

4.Please provide confusion matrices for each validation cohort at the selected threshold.

5.Please clarify how the Mayo ATTR-CM score was calculated when variables were missing.

6.Please clarify whether the external validation site used the same DPD acquisition, reconstruction, reporting, and grading protocols as the development site.

7.Please avoid wording such as "life-saving treatments" unless specifically justified, as it may sound promotional, suggest "guideline-directed".

8.The phrase "promises to address diagnostic delays and improve outcomes" should be softened to "may help address diagnostic delays, pending prospective evaluation".

9.Please correct typographical errors, including "cardac" and inconsistent capitalisation/formatting of HFpEF.

10.Please provide more detail on the feature selection process, including the exact final 13 variables used in the model.

11.Please include a clearer explanation of how the app handles missing values, including whether a fully missing input vector is allowed by design.

12. Please ensure ATTR-CM or CM is defined in full at first use.

**********

what does this mean?). If published, this will include your full peer review and any attached files.

**Do you want your identity to be public for this peer review?**  If you choose “no”, your identity will remain anonymous but your review may still be made public.

For information about this choice, including consent withdrawal, please see our Privacy Policy

Reviewer #1: No

Reviewer #2: **Yes:** Dr Ahmed El-Medany MBChB MSc MRCP FHEA

**********

**Figure resubmission:**

**Reproducibility:**

To enhance the reproducibility of your results, we recommend that authors of applicable studies deposit laboratory protocols in protocols.io, where a protocol can be assigned its own identifier (DOI) such that it can be cited independently in the future. Additionally, PLOS ONE offers an option to publish peer-reviewed clinical study protocols. Read more information on sharing protocols at https://plos.org/protocols?utm_medium=editorial-email&utm_source=authorletters&utm_campaign=protocols

# Author comment: Author response to Decision Letter 1

**DOI:** 10.1371/journal.pdig.0001637.r002

17 Jun 2026

Attachment Submitted filename: Response_to_Reviewers.docx

# Peer review: Decision Letter 1

**DOI:** 10.1371/journal.pdig.0001637.r003

**Authors:** Peter H Charlton

19 Jul 2026

Screening for patients at risk for cardiac amyloidosis via electronic health records: A multicenter machine learning development and validation study

PDIG-D-26-00090R1

Dear Mr. Nitsche,

We are pleased to inform you that your manuscript 'Screening for patients at risk for cardiac amyloidosis via electronic health records: A multicenter machine learning development and validation study' has been provisionally accepted for publication in PLOS Digital Health.

Before your manuscript can be formally accepted you will need to complete some formatting changes, which you will receive in a follow-up email from a member of our team.

Please note that your manuscript will not be scheduled for publication until you have made the required changes, so a swift response is appreciated.

IMPORTANT: The editorial review process is now complete. PLOS will only permit corrections to spelling, formatting or significant scientific errors from this point onwards. Requests for major changes, or any which affect the scientific understanding of your work, will cause delays to the publication date of your manuscript.

If your institution or institutions have a press office, please notify them about your upcoming paper to help maximize its impact. If they'll be preparing press materials, please inform our press team as soon as possible -- no later than 48 hours after receiving the formal acceptance. Your manuscript will remain under strict press embargo until 2 pm Eastern Time on the date of publication. For more information, please contact digitalhealth@plos.org.

Thank you again for supporting Open Access publishing; we are looking forward to publishing your work in PLOS Digital Health.

Best regards,

Peter H Charlton, MEng, PhD

Section Editor

PLOS Digital Health

***********************************************************

**Additional Editor Comments (if provided):**

**Reviewer Comments (if any, and for reference):**

Reviewer's Responses to Questions

**Comments to the Author**

Reviewer #1: All comments have been addressed

Reviewer #2: All comments have been addressed

**********

publication criteria?

Reviewer #1: Yes

Reviewer #2: Yes

**********

3. Has the statistical analysis been performed appropriately and rigorously?

Reviewer #1: Yes

Reviewer #2: Yes

**********

4. Have the authors made all data underlying the findings in their manuscript fully available (please refer to the Data Availability Statement at the start of the manuscript PDF file)?

The PLOS Data policy

Reviewer #1: Yes

Reviewer #2: Yes

**********

5. Is the manuscript presented in an intelligible fashion and written in standard English?

Reviewer #1: Yes

Reviewer #2: Yes

**********

Reviewer #1: Thank you for clear answers to my comments.

Reviewer #2: Many thanks for your hard work and thorough responses to my comments. The manuscript is in an excellent state now and I am satisfied with your changes. Good luck with the rest of the publication process.

**********

what does this mean?). If published, this will include your full peer review and any attached files.

**Do you want your identity to be public for this peer review?**  If you choose “no”, your identity will remain anonymous but your review may still be made public.

For information about this choice, including consent withdrawal, please see our Privacy Policy

Reviewer #1: No

Reviewer #2: **Yes:** Dr Ahmed El-Medany

**********
